# Supplementary material for: Varying strength of selection contributes to the intragenomic diversity of rRNA genes
Source: Nat Commun. 2022 Nov 25;13:7245. doi: 10.1038/s41467-022-34989-w (PMC9700816; doi:10.1038/s41467-022-34989-w)
Supplement: Supplementary file 1 — Supplementary Information [file 41467_2022_34989_MOESM1_ESM.pdf]

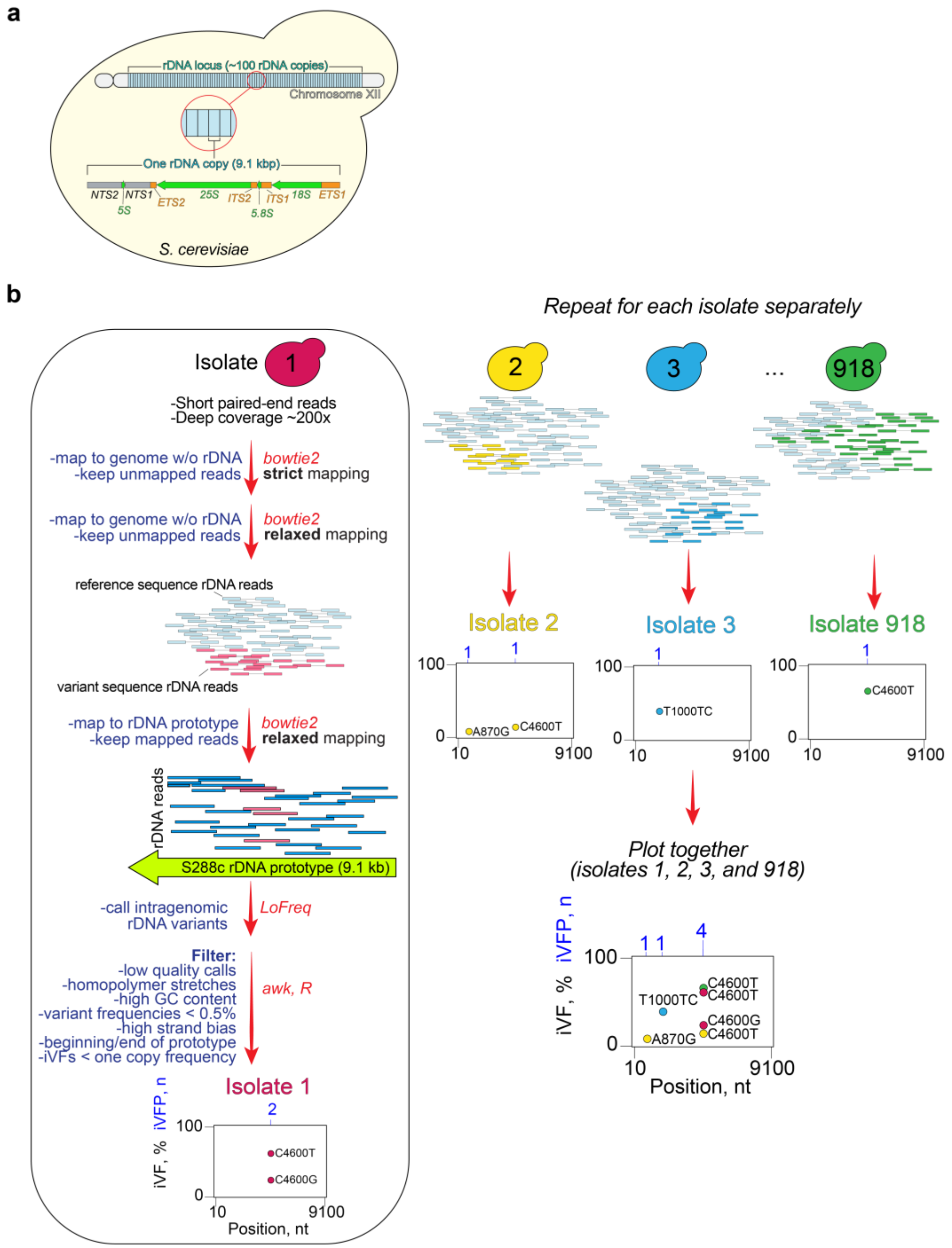

Supplementary Figure 1. Pipeline overview. a, organization of the rDNA locus in *S. cerevisiae*. About 100 rDNA copies are encoded in a head-to-tail fashion on chromosome XII. Each rDNA copy is 9.1 kbp long and contains rRNA genes (*25S*, *18S*, *5.8S*, *5S*; green), gene regulatory elements (*NTS1*, *NTS2*; grey), and transcribed elements that control rRNA processing (*ETS1*, *ETS2*, *ITS1*, *ITS2*; orange). **b**, bioinformatics pipeline described in Methods. The pipeline was run on each isolate separately.

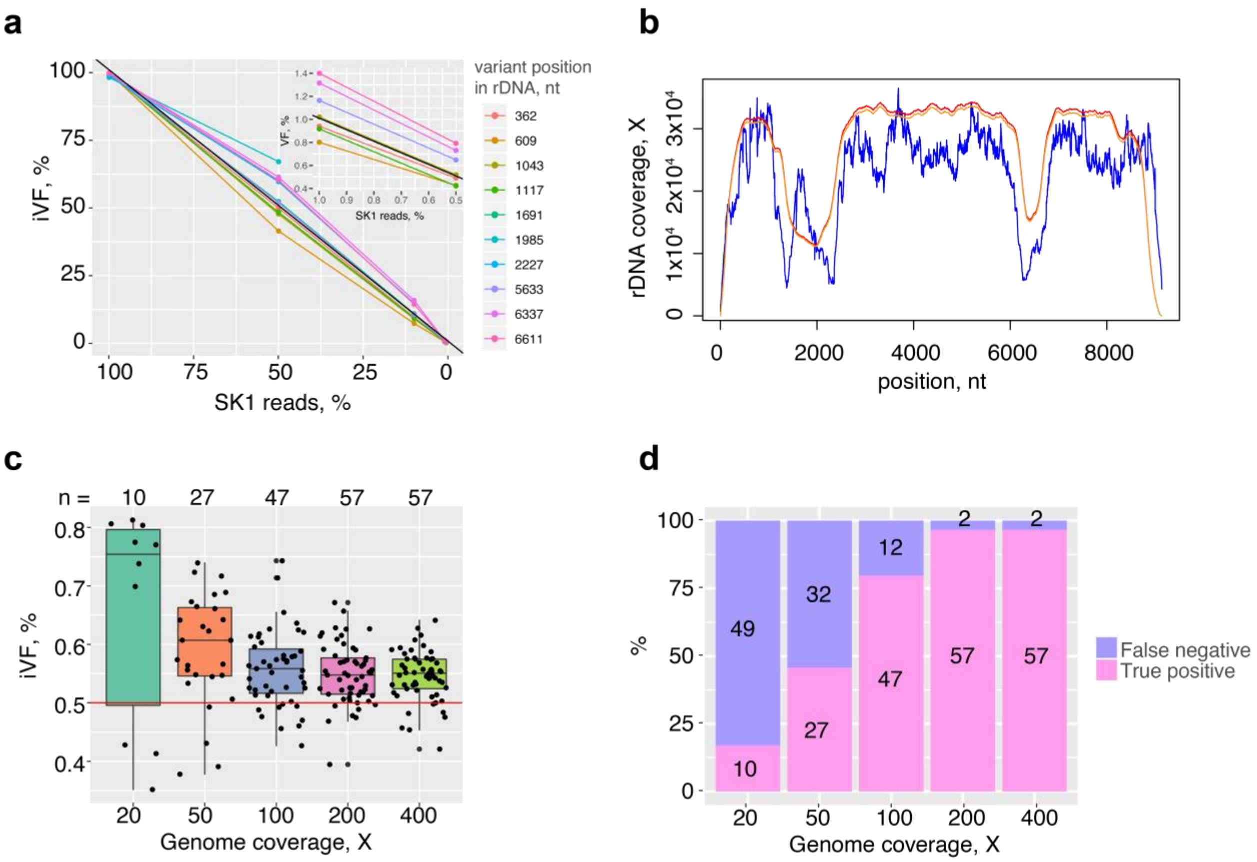

Supplementary Figure 2. Pipeline benchmarking. a, *in silico* spiking of S288c read data with 10 highly confident SNVs from another lab strain (SK1) across the rDNA prototype (positions are shown in the legend). The black diagonal represents true iVF values. *inset* - VFs from 1 to 0.5%. **b**, 200X genomic read coverage across the rDNA copy from S288c experimental data (blue), for a simulated dataset with true priors before (red) or after (orange) running the bioinformatics pipeline. **c**, pipeline performance on simulated datasets of different genomic coverage (20X-400X) but with the same variant alleles (all iVF = 0.5%, red line; dots are true positive variants). For each box, the center line is the median, box limits are 25th and 75th percentiles, and the whiskers extend to ± 1.5xIQR (interquartile range). N is the number of true positives. **d**, pipeline specificity as a function of coverage. Absolute values are indicated in the plot. No false positives were detected (see Methods). Source data are provided as a Source Data file.

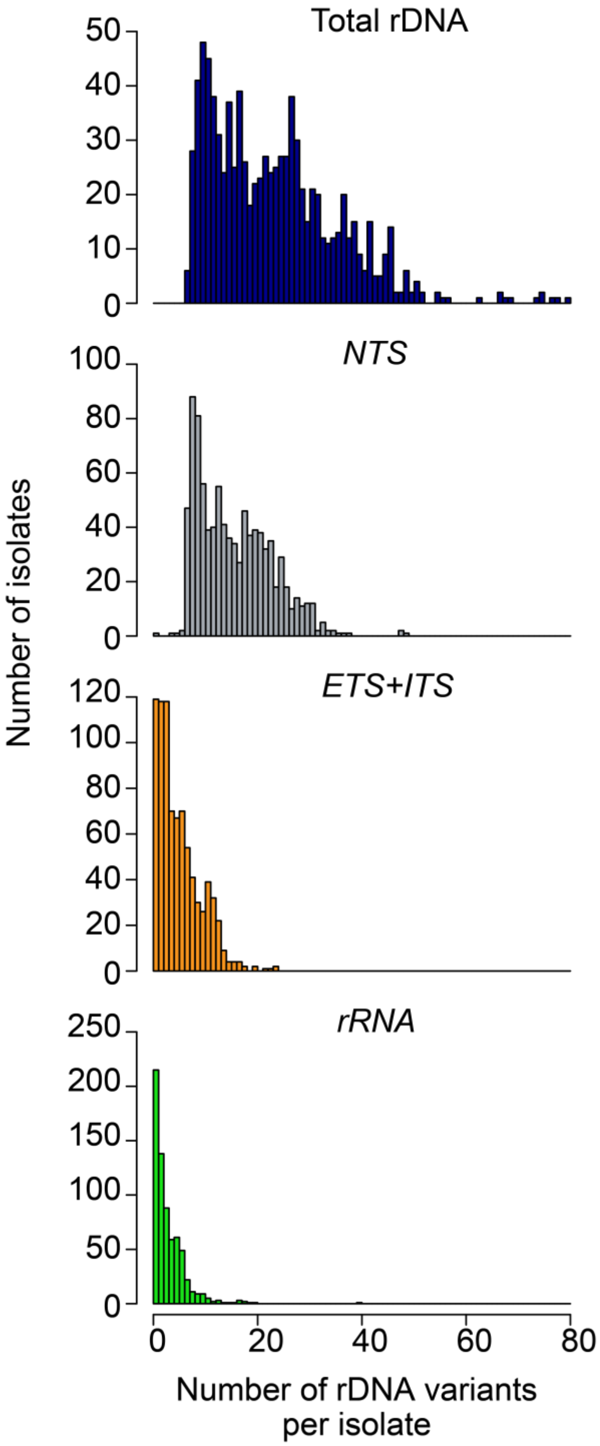

Supplementary Figure 3. Distribution of the number of rDNA variants per isolate. NTS – *NTS1* and *NTS2*; ETS+ITS – *ETS1*, *ETS2*, *ITS1* and *ITS2*; rRNA – rRNA genes (*5S*, *5.8S*, *18S* and *25S*). Source data are provided as a Source Data file.

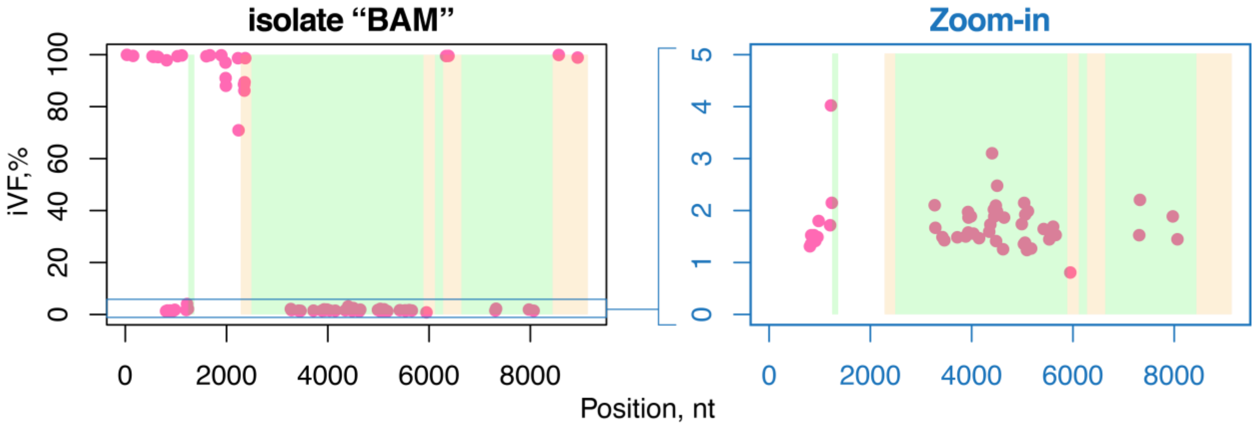

Supplementary Figure 4. Distribution of variants and their iVFs in the "BAM" isolate. Each dot is a variant. Source data are provided as a Source Data file.

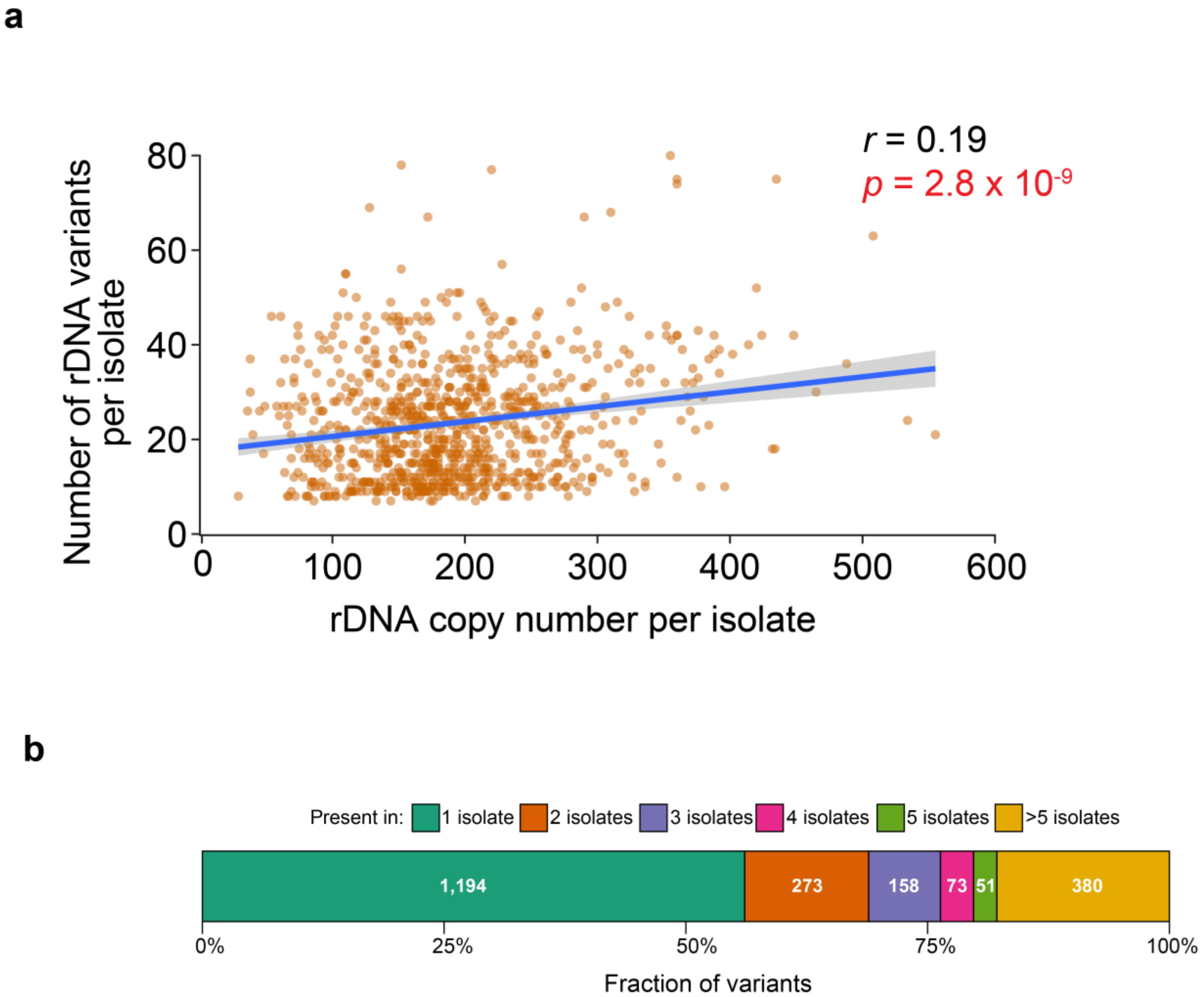

Supplementary Figure 5. **a,** correlation between the number of rDNA variants and the total number of rDNA copies per isolate. *R* – Pearson correlation coefficient. Each dot represents an isolate. The regression line (blue) is plotted with linear regression model. The gray shading is a 95% confidence interval (CI). Significance test: two-sided Pearson's product-moment correlation, t(915) = 6, p = 2.8x10^{-9}, r = 0.19, 95% CI [0.1, 0.26].. **b**, sharedness of variants across isolates. Numbers inside the bars indicate the number of variants in each category. Source data are provided as a Source Data file.

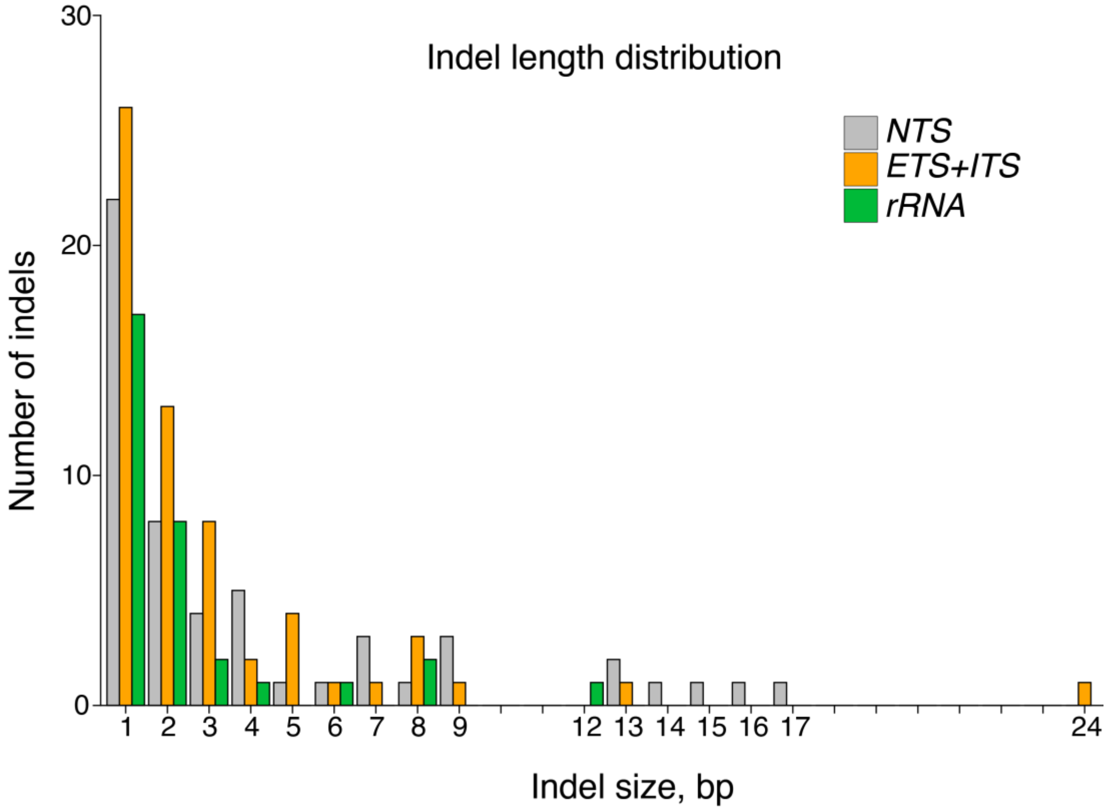

Supplementary Figure 6. Indel length distribution across rDNA. The number of indels across all isolates is shown as a function of indel size. Source data are provided as a Source Data file.

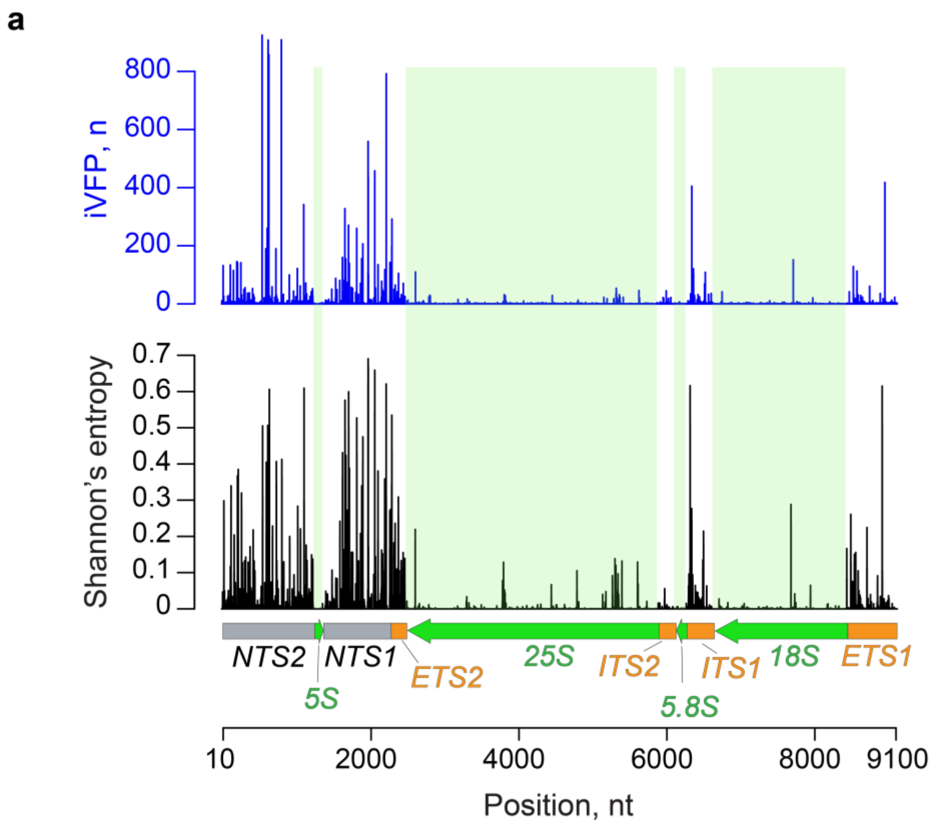

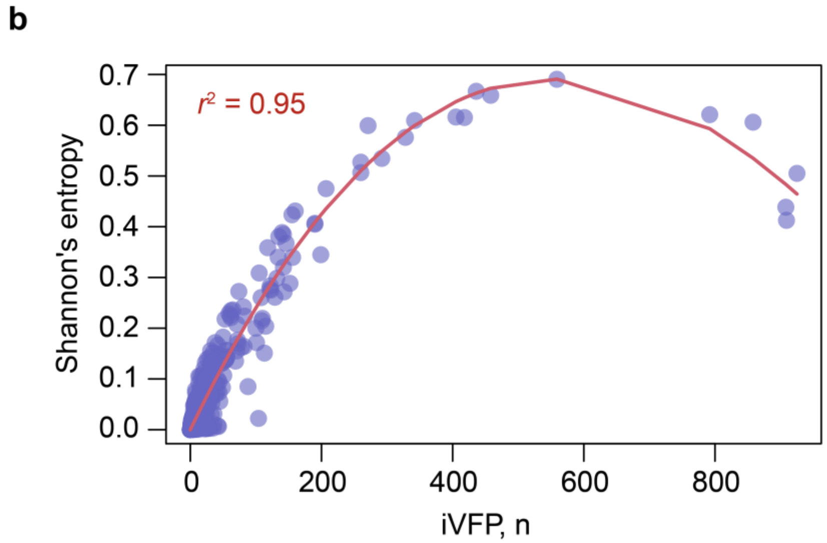

Supplementary Figure 7. a, Sequence diversity at each nucleotide (Shannon's entropy) based on iVFPs calculated for each rDNA position. Source data are provided in Supplementary Data 2. **b**, correlation between entropy and the number of iVFPs at a given position. Each dot represents a nucleotide position in rDNA. Best-fit polynomial is shown in red. Source data are provided as a Source Data file.

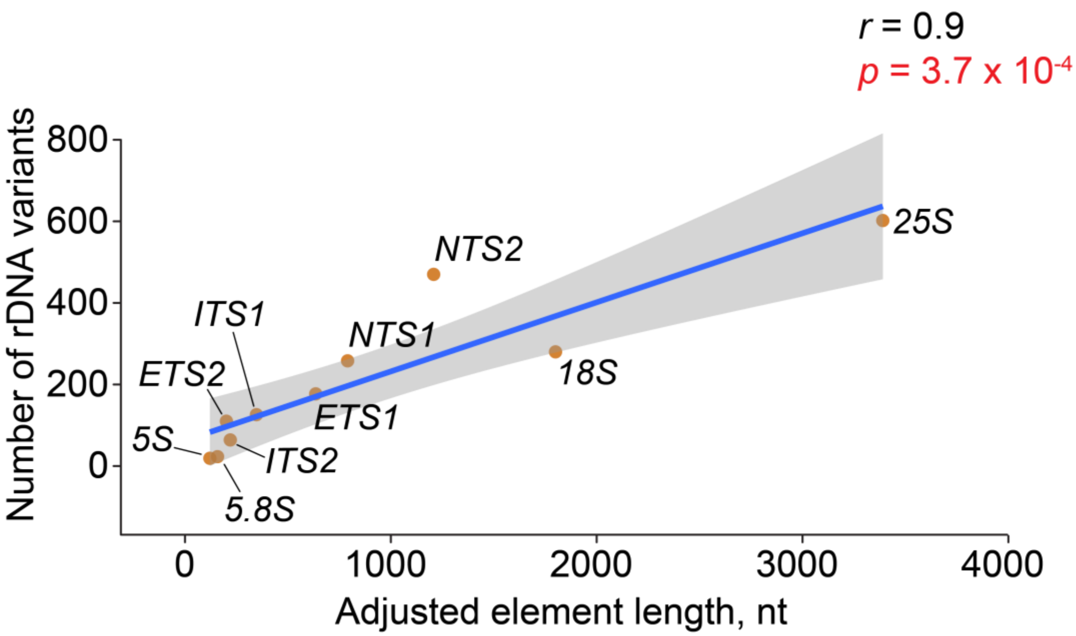

Supplementary Figure 8. Correlation between the number of rDNA variants per element and the length of the corresponding element. *R* – Pearson correlation coefficient. Brown dots represent different elements. The regression line (blue) is plotted with linear regression model. The gray shading is a 95% CI. Significance test: two-sided Pearson's product-moment correlation, t(8) = 5.88, $p = 3.7x10^{-4}$, $r = 0.9$, 95% CI [0.63, 0.98].. Adjusted element lengths are due to filtering (see Methods). Source data are provided as a Source Data file.

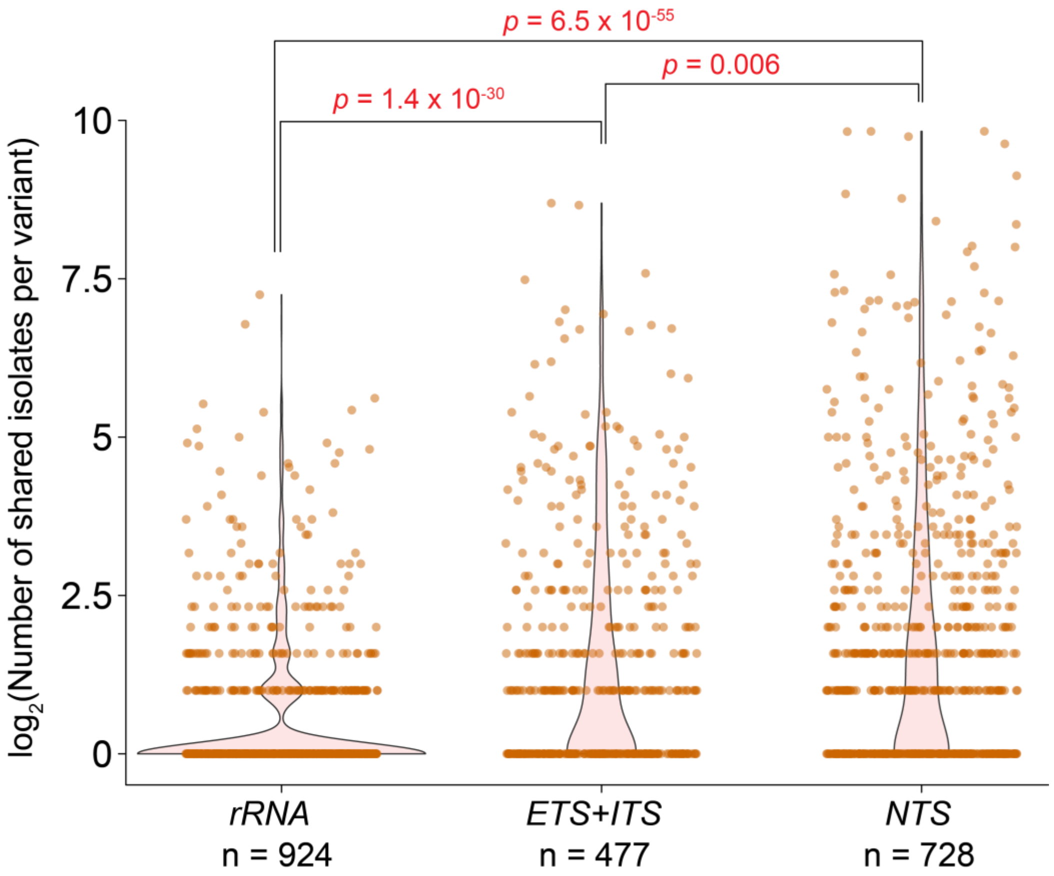

Supplementary Figure 9. Sharedness of variants across isolates is stratified by rDNA elements. n - total number of variants (shown as dots)in the group. Y-axis is shown in log2 scale. Significance test: two-sided pairwise Wilcoxon rank sum test with Benjamini–Hochberg correction; rRNA vs NTS: W = 202,065, p = 6.5x10^{-55}, location shift = -0.99, 95% CI [-1.0, -0.99]; rRNA vs ETS+ITS: W = 149,869, p = 1.4x10^{-30}, location shift = -1.32x10^{-5} , 95% CI [-0.99, -7.1x10^{-5}]; ETS+ITS vs NTS: W = 158,028, p = 0.006, location shift = -3.07x10^{-6}, , 95% CI [-9.9x10^{-6}, -1.3x10^{-5}]. Source data are provided as a Source Data file.

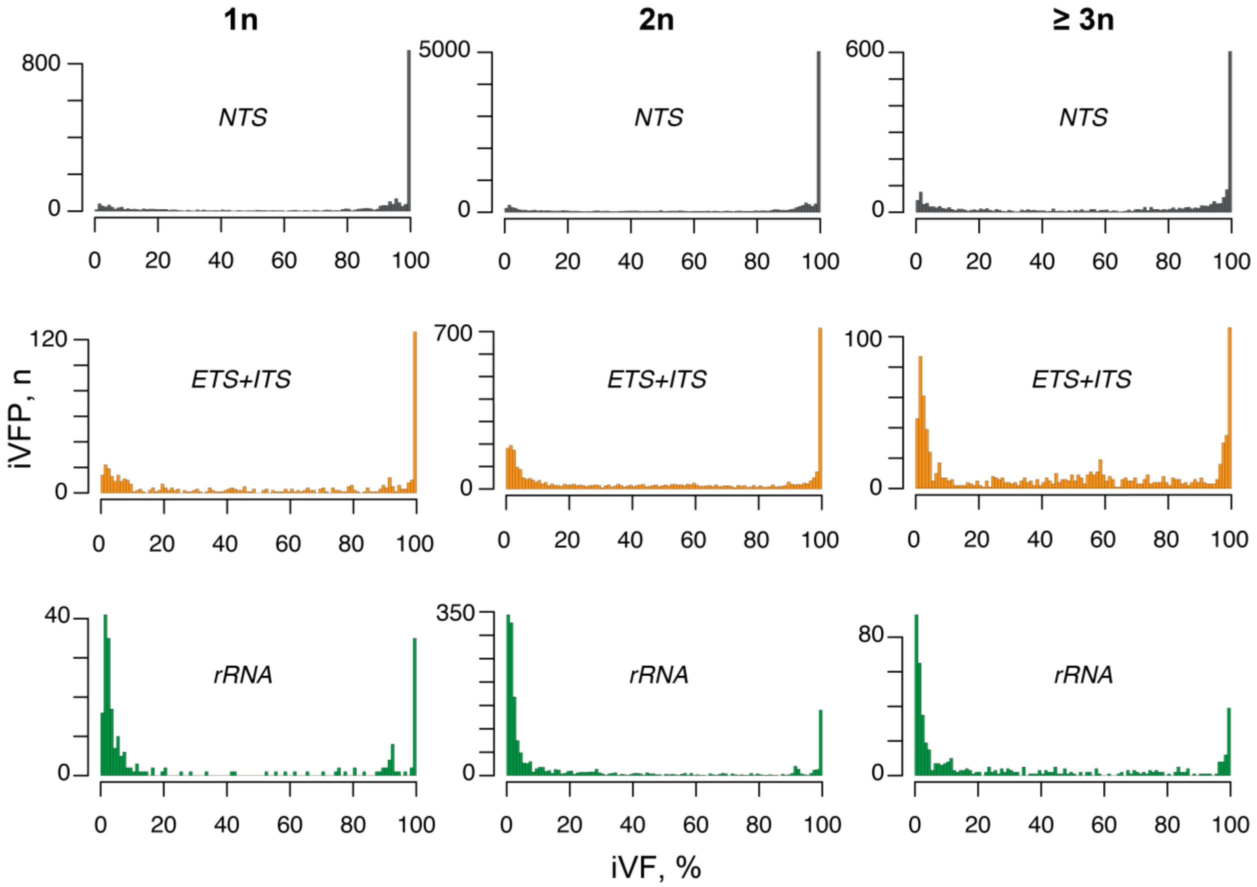

Supplementary Figure 10. Distribution of iVFPs separated by rDNA elements and isolates' ploidy. 1n - haploid, 2n - diploid, >= 3n - polyploid isolates. Source data are provided as a Source Data file.

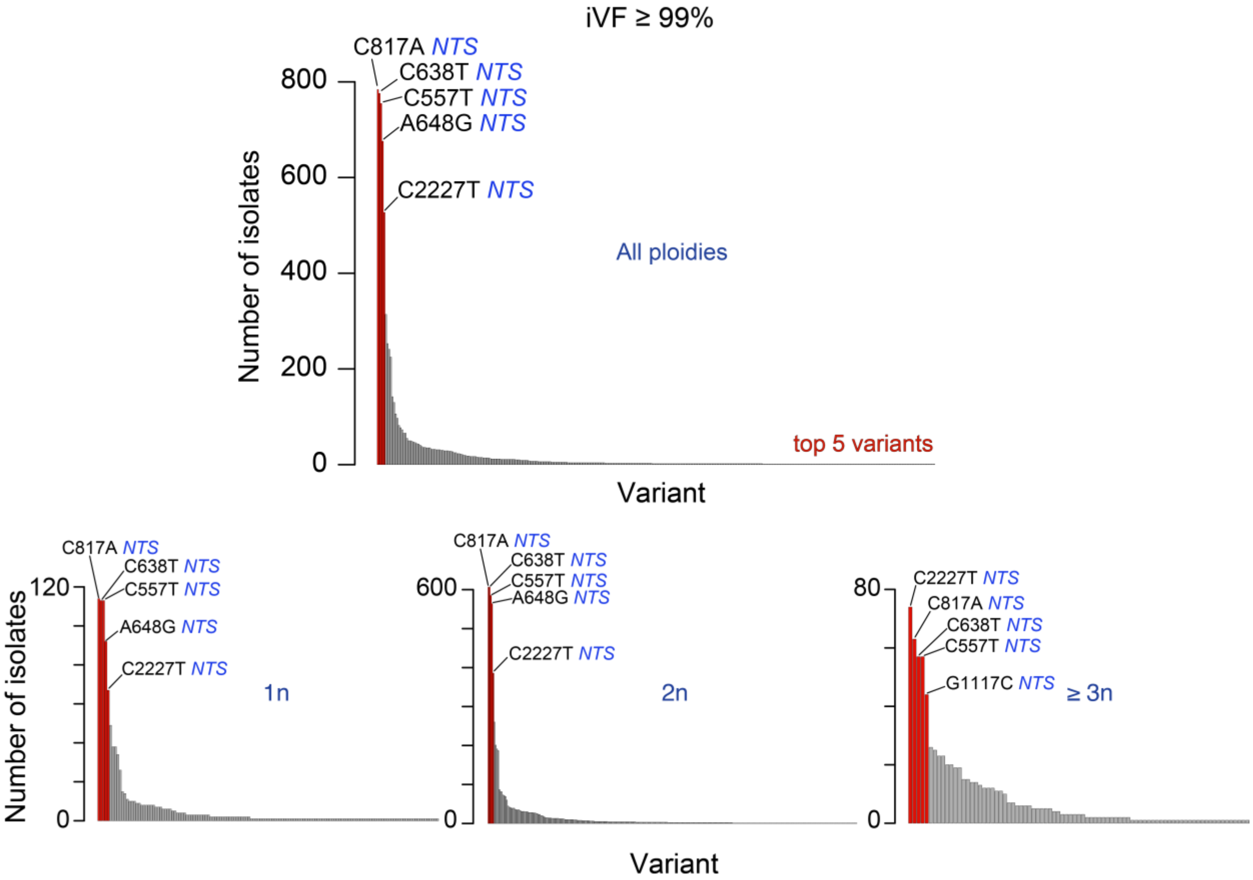

Supplementary Figure 11. Distribution of variants with iVF ≥ 99% across isolates. Each bar represents a variant, and the height of the bar (y-axis) shows how many isolates contain the variant at iVF ≥ 99%. The plots are shown for all isolates ("All ploidies", *top*), and separated by ploidy (*bottom*). The most common variants were all located in *NTS*. Source data are provided as a Source Data file.

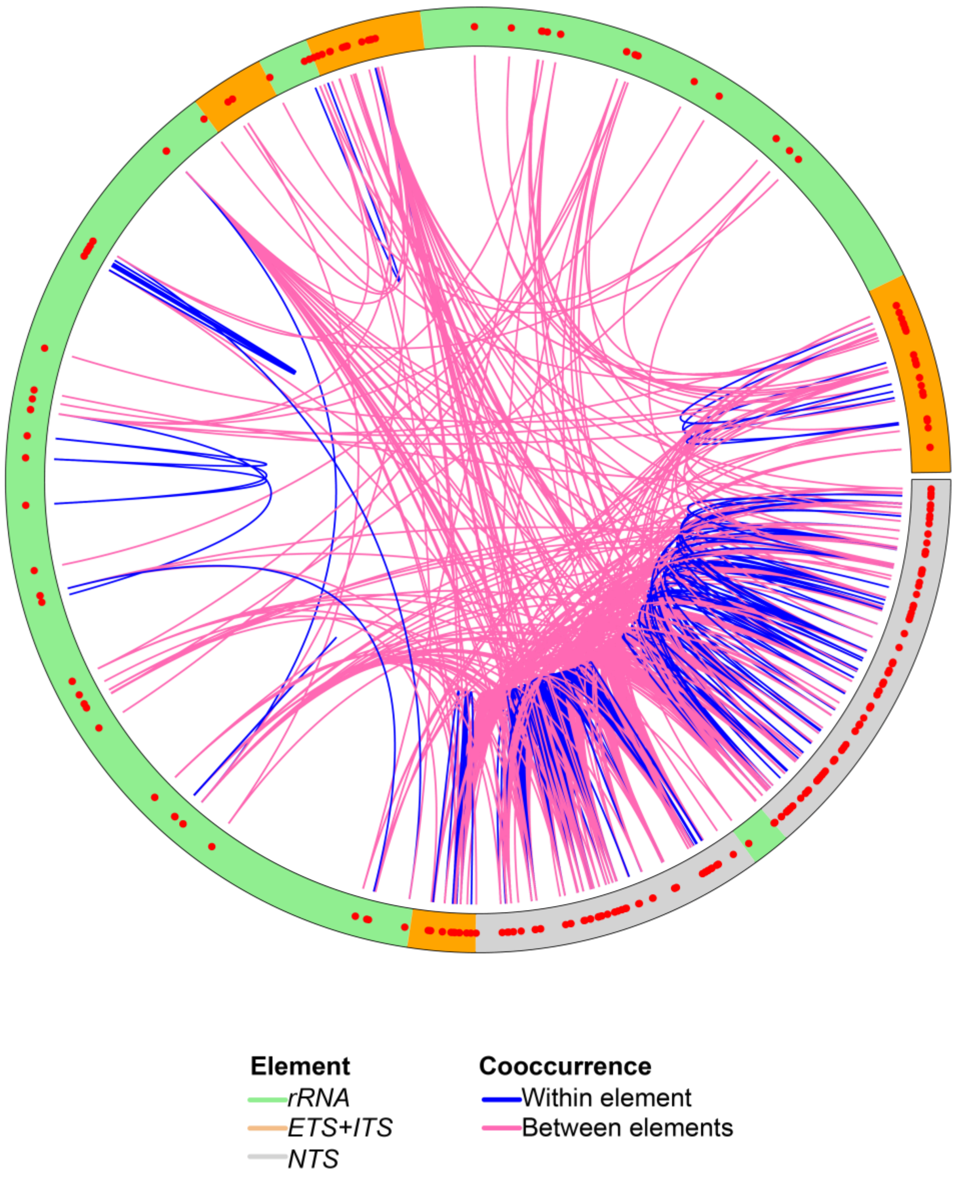

Supplementary Figure 12. Variants with consistently similar iVFs. Cooccurrence was calculated on all isolates based on Euclidean distances (see Methods) between iVFs for every pair of variants within an isolate. An rDNA copy is shown as a circle with colors corresponding to different elements (green – *rRNA;* orange – *ETS1*, *ETS2*, *ITS1* and *ITS2*; grey – *NTS1* and *NTS2*) and variants are shown as red dots. Blue and pink lines indicate variant pairs within or between rDNA elements, respectively. A variant can be connected to multiple other variants. Source data are provided in Supplementary Data 3.

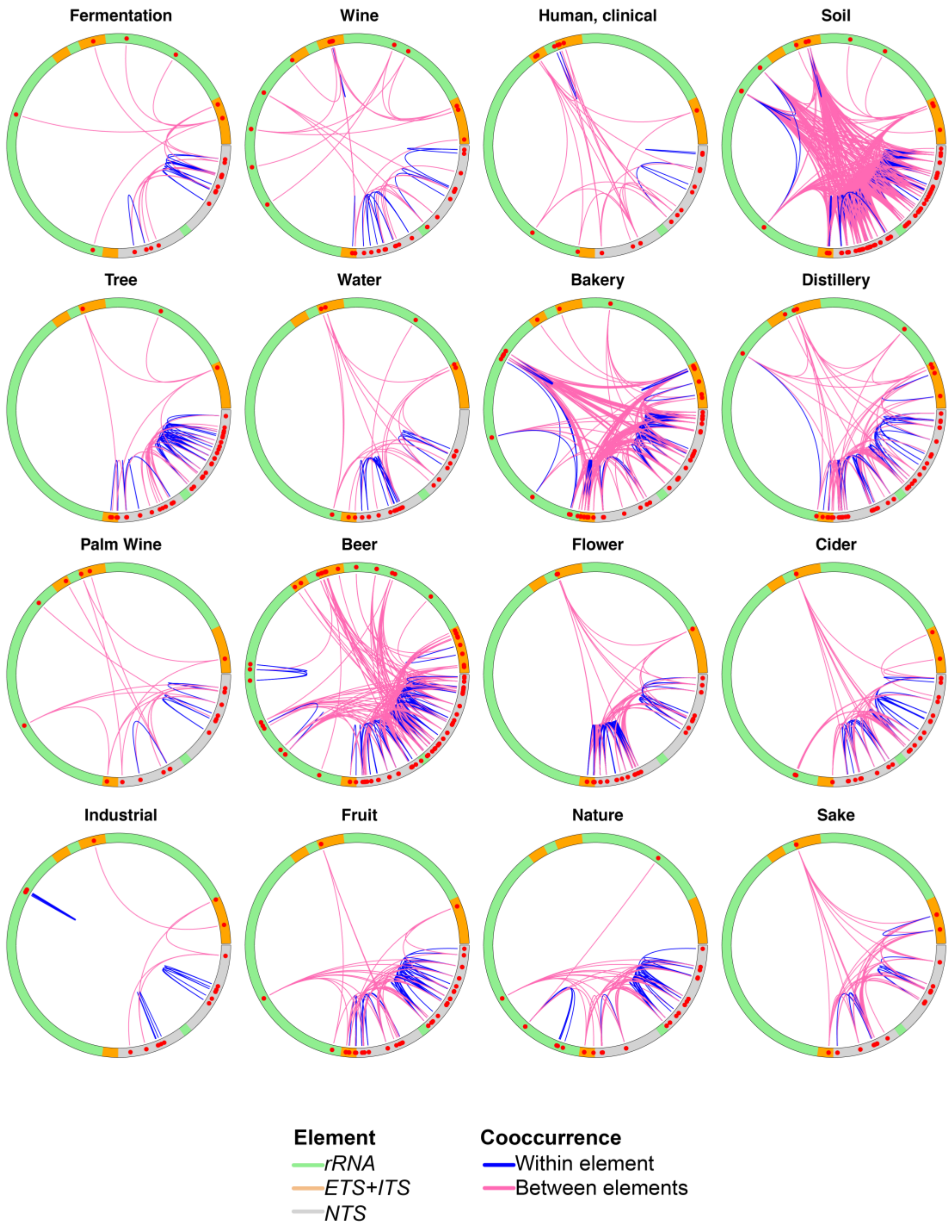

Supplementary Figure 13. Variants with consistently similar iVFs in different ecological niches. Same as **Supplementary Figure 12** but performed within each ecological niche individually. Source data are provided in Supplementary Data 4.

a

		Variants 1	2	3	4	5	
	rDNA nomenclature	**5322G;**	**5338C;**	**5343A;**	**5349T;**	**5360C**	
	25S nomenclature	**565C;**	**549G;**	**544U;**	**538A;**	**527G**	
Industrial	ABM	>99%	>99%	>99%	>99%	>99%	iVF
	AHL	>99%	>99%	>99%	>99%	>99%	
	AHM	>99%	>99%	>99%	>99%	>99%	
	ATQ	>99%	>99%	>99%	>99%	>99%	
	AQD	>99%	>99%	>99%	>99%	>99%	
	BRK	>99%	>99%	>99%	>99%	>99%	
	AQA	27.9%	28.3%	28.3%	28.3%	28.0%	

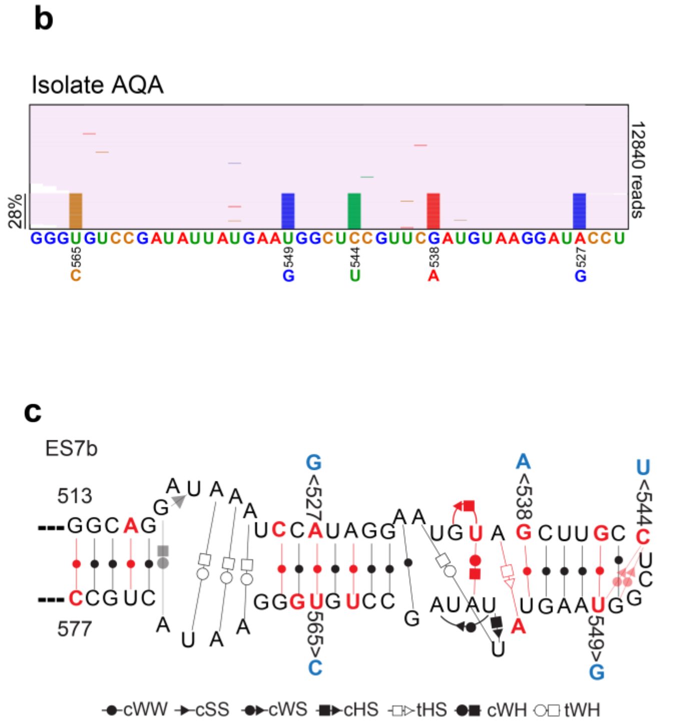

Supplementary Figure 14. Five variants cooccur in rDNA copies in several isolates. a, iVFs of the five variants in *25S* among seven isolates. **b**, continuous rDNA reads of isolate "AQA" mapped to *25S*. All the variants are in the same reads and only a subset of rDNA copies (28%) contain the variants in the genome. Source data are provided as a Source Data file. **c**, localization of the variants (blue) in the two-dimensional context of the hairpin from expansion segment ES7b. Variants that were detected across all the isolates in this region are shown in red. Types of interaction are described below the structure; cWW: cis-Watson-Crick/Watson-Crick, cSS: cis-Sugar/Sugar, cWS: cis-Watson-Crick/Sugar, cHS: cis-Hoogsteen/Sugar, tHS: trans-Hoogsteen/Sugar, cWH: cis-Watson-Crick/Hoogsteen, tWH: trans-Watson-Crick/Hoogsteen.

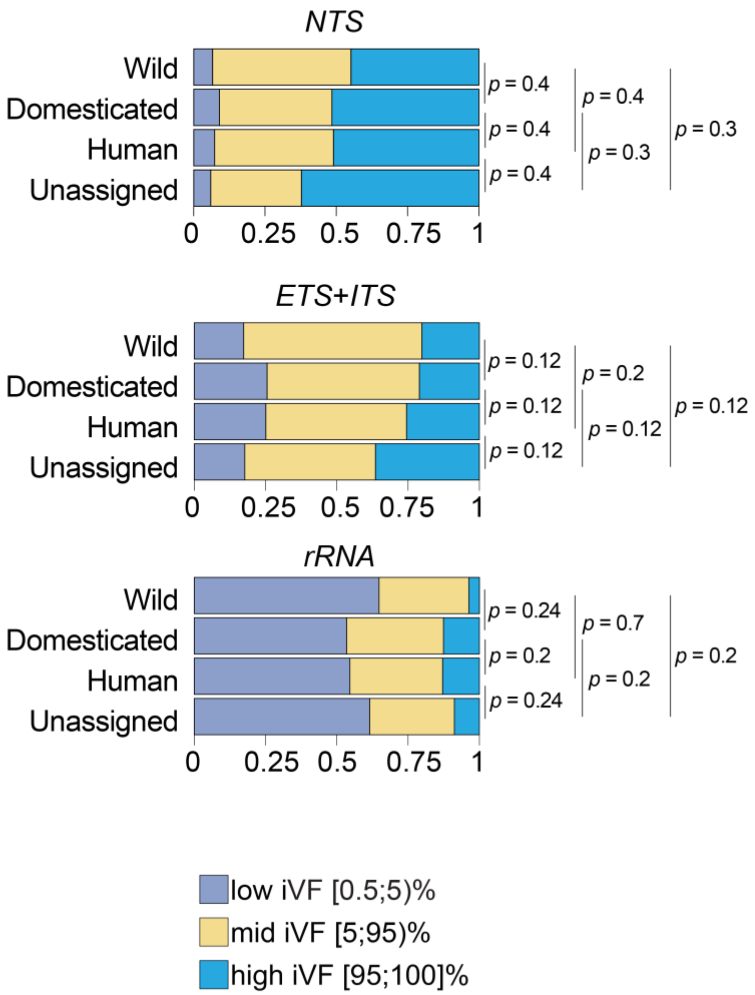

Supplementary Figure 15. iVFP distribution based on rDNA genic elements in different ecological groups from [1]. *P* – values were calculated by using two-sided Wilcoxon rank sum test with Benjamini-Hochberg correction for multiple hypothesis testing. Source data are provided as a Source Data file.

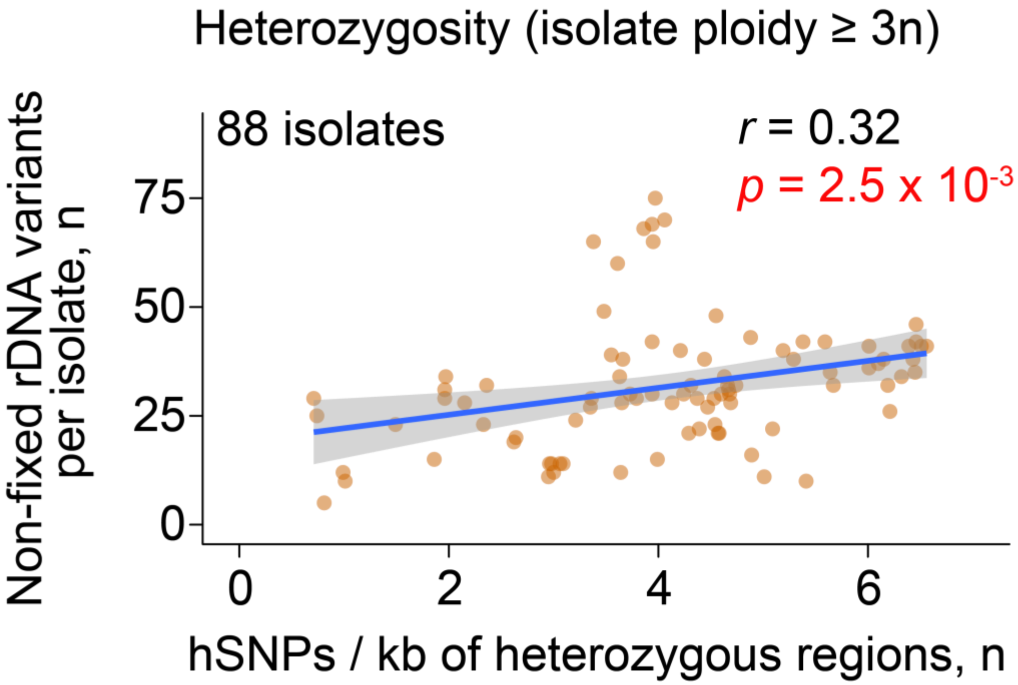

Supplementary Figure 16. Relationship between heterozygosity of the rDNA and the rest of the genome in polyploid (3n-5n) isolates. Correlation between the number of rDNA variants and the number of heterozygous SNPs per heterozygous region of the genome (excluding rDNA; from [2]). Pearson correlation coefficient *R* is shown. Each dot represents an isolate. The regression line (blue) is plotted with linear regression model. The gray shading is a 95% CI. Significance test: two-sided Pearson's product-moment correlation, t(86) = 3.11, $p = 2.5 \times 10^{-3}$, $r = 0.32$, 95% CI [0.12, 0.49]. Source data are provided as a Source Data file.

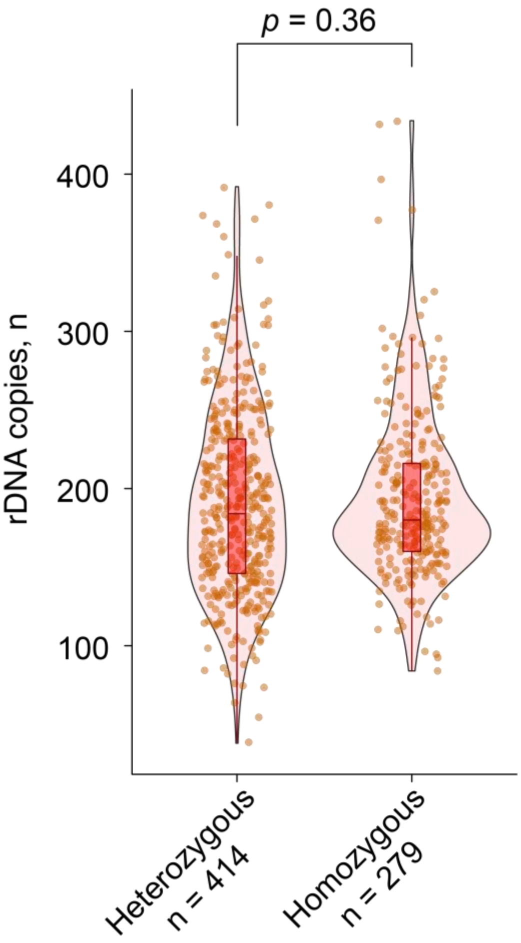

Supplementary Figure 17. Number of rDNA copies in heterozygous and homozygous diploid isolates analyzed in **Fig. 3** (main text). n indicates the number of isolates (shown as dots) in each group. *P*-value was calculated using two-sided Wilcoxon rank sum test. Source data are provided as a Source Data file.

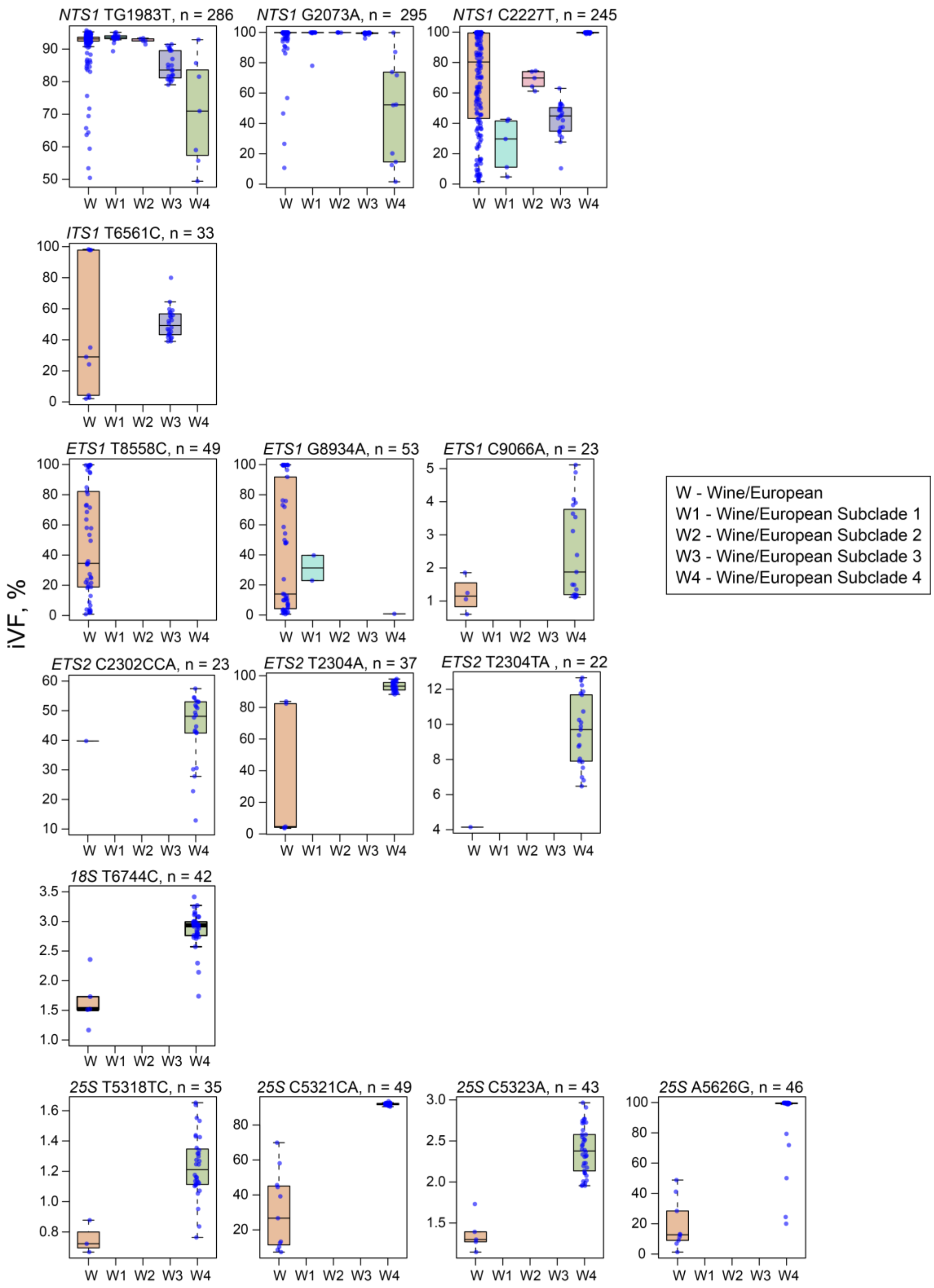

Supplementary Figure 18. Distribution of some of the most represented iVFPs in the Wine/European subclades. n indicates the total number of iVFPs (shown as dots) for each variant across subclades. Source data are provided as a Source Data file.

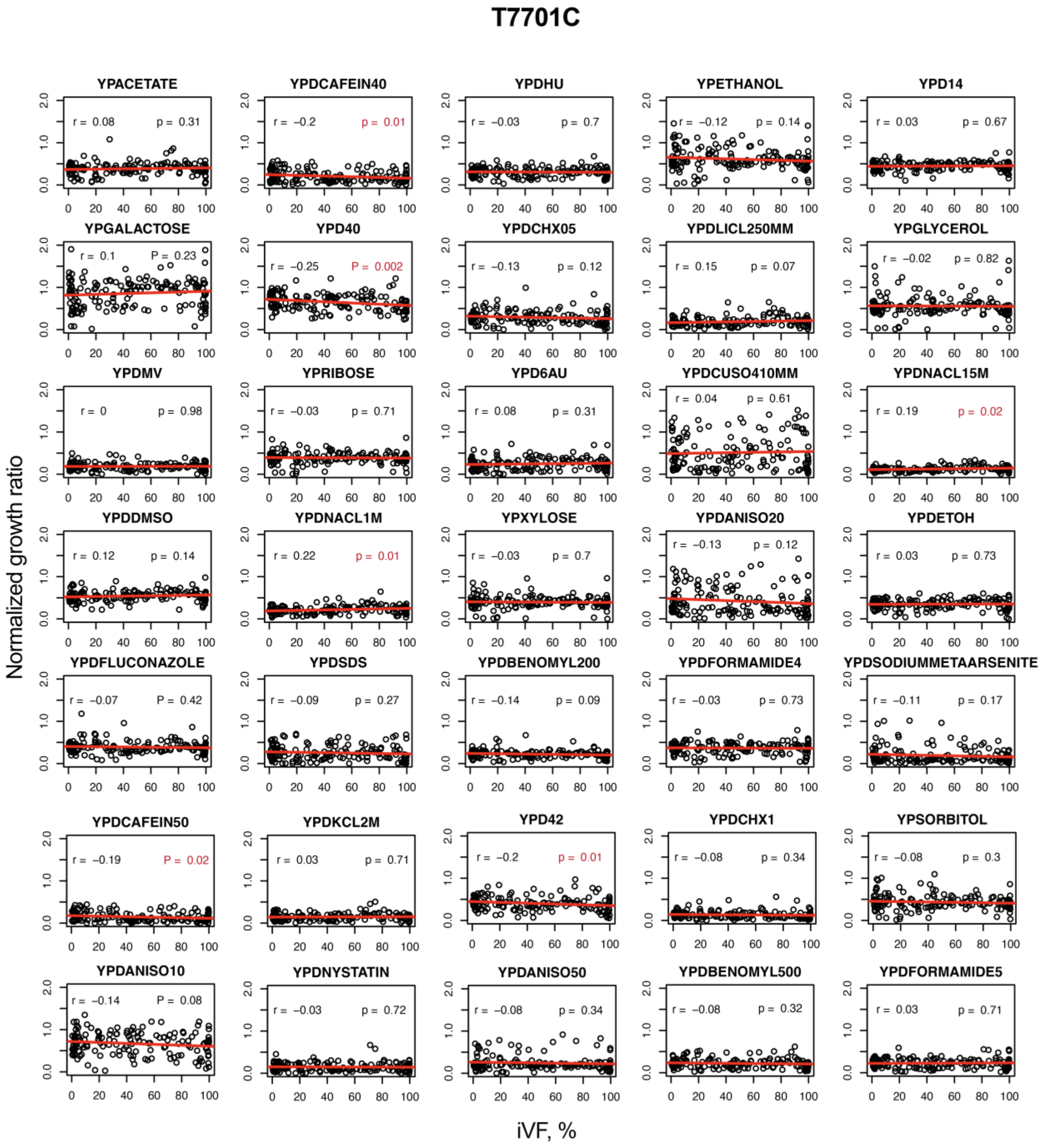

Supplementary Figure 19. Correlation between fitness and variant's iVFs for variant T7701C. Stress conditions and their fitness values (growth ratio normalized to a non-stressed growth condition) are taken from [2]. The dots represent isolates. For each plot, two-sided Pearson's product-moment correlation (not adjusted for multiple hypothesis testing) was performed as a significance test. Pearson correlation coefficient R and a P-value are shown. Source data are provided as a Source Data file

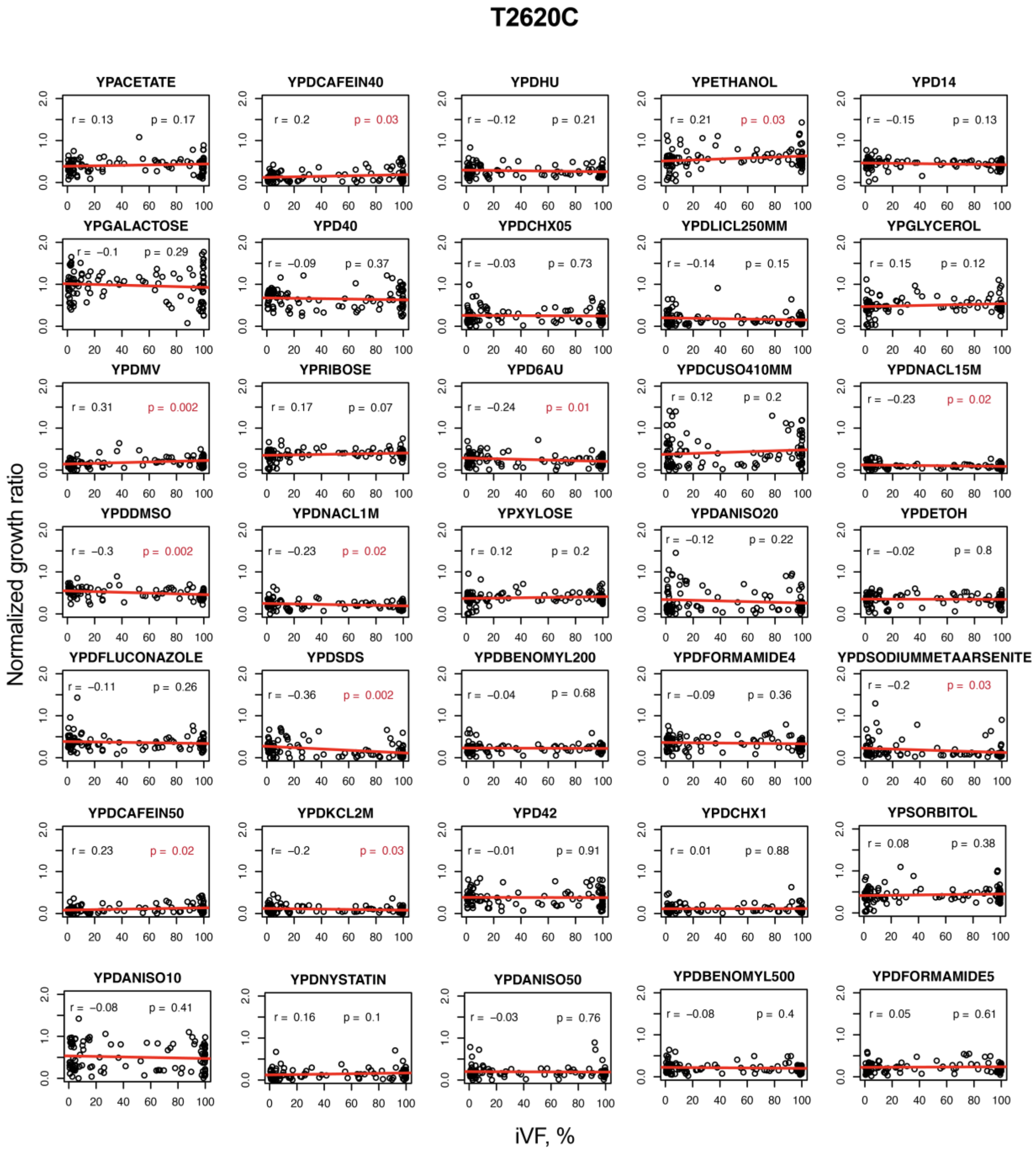

Supplementary Figure 20. Correlation between fitness and variant's iVFs for variant T2620C. Stress conditions and their fitness values (growth ratio normalized to a non-stressed growth condition) are taken from [2]. The dots represent isolates. For each plot, two-sided Pearson's product-moment correlation (not adjusted for multiple hypothesis testing) was performed as a significance test. Pearson correlation coefficient R and a P-value are shown. Source data are provided as a Source Data file.

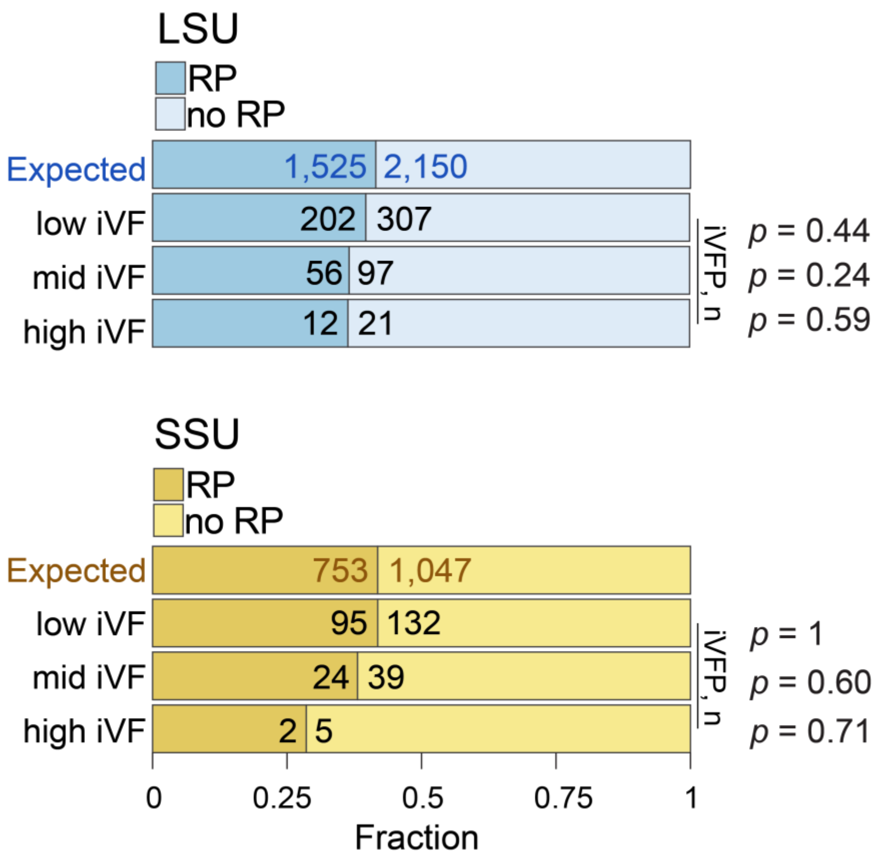

Supplementary Figure 21. Distribution of VFPs that are involved in rRNA-ribosomal protein (RP) interactions. LSU – large subunit, SSU – small subunit. P-values are calculated using two-sided Fisher's exact test with Benjamini-Hochberg correction for multiple hypothesis testing. Source data are provided as a Source Data file.

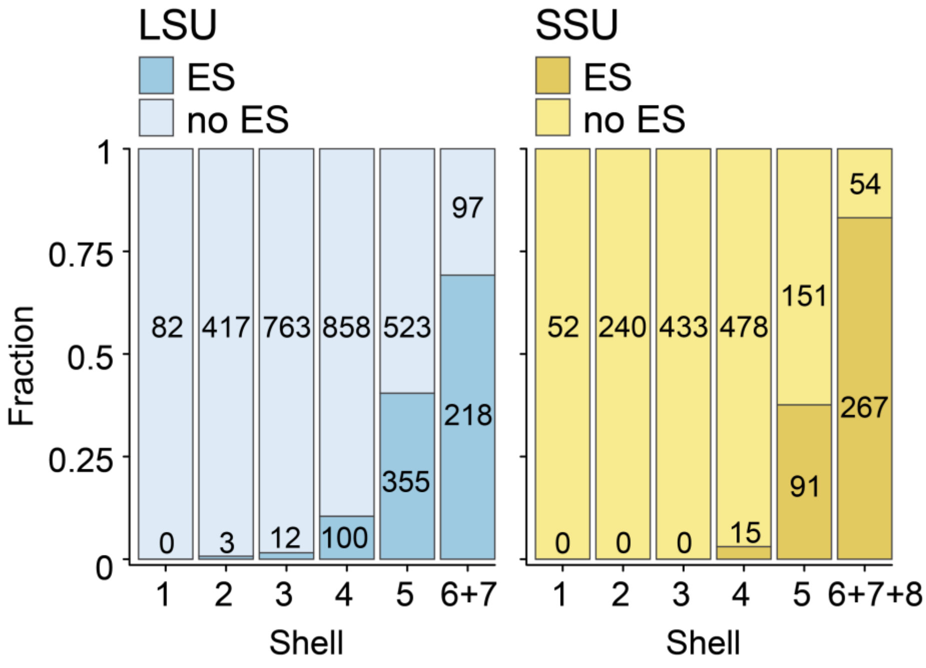

Supplementary Figure 22. Distribution of nucleotides from expansion segments (ES) across shells. "no ES" – nucleotides outside ES. Source data are provided as a Source Data file.

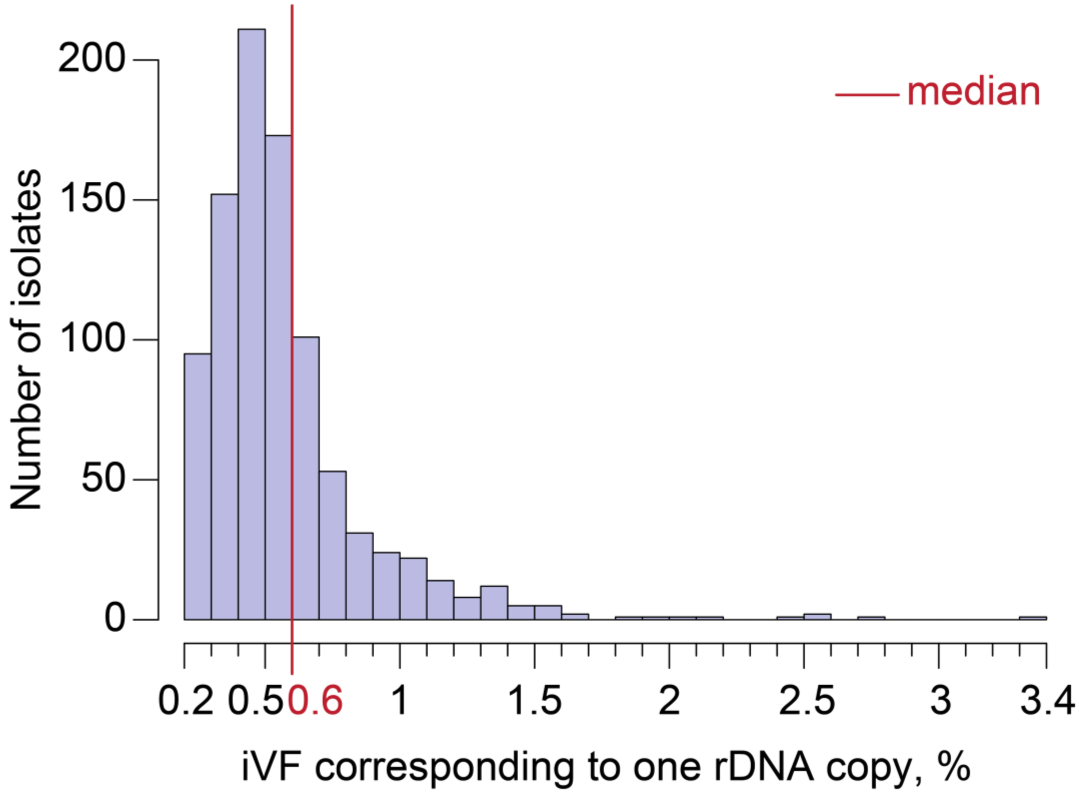

Supplementary Figure 23. Distribution of iVFs that correspond to one rDNA copy. Each iVF was calculated for every isolate from its total rDNA copy number. The red line indicates the median. Source data are provided as a Source Data file.

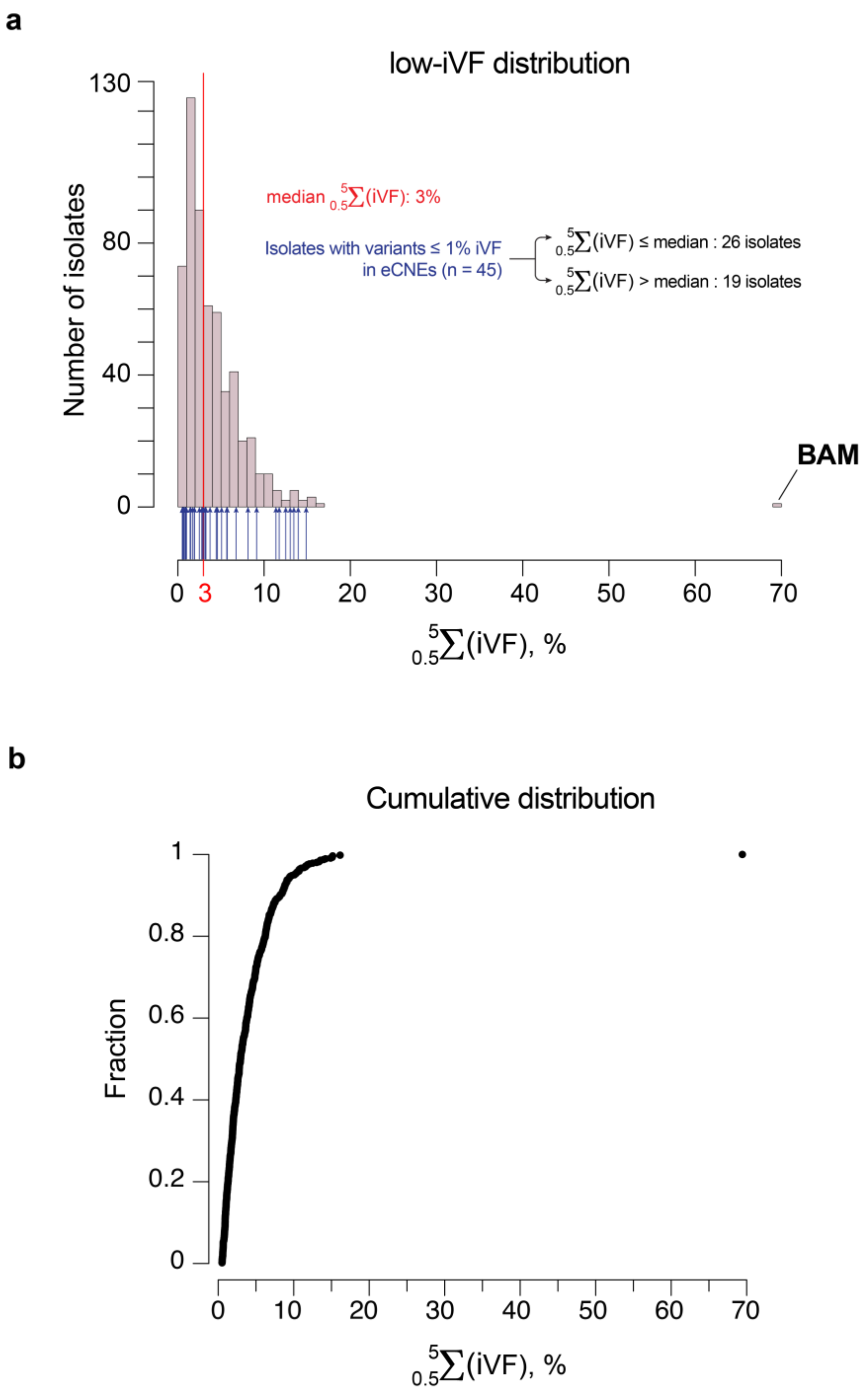

Supplementary Figure 24. a, distribution of sums of low iVFs in coding regions (*5S*, *5.8S*, *18S*, *25S*) across all isolates. Green arrows indicate the presence of isolates with less than 1% of variants in CNEs. The median is shown in red. The "BAM" isolate is indicated in the plot. **b** – cumulative distribution of the same dataset. Source data are provided as a Source Data file.

a

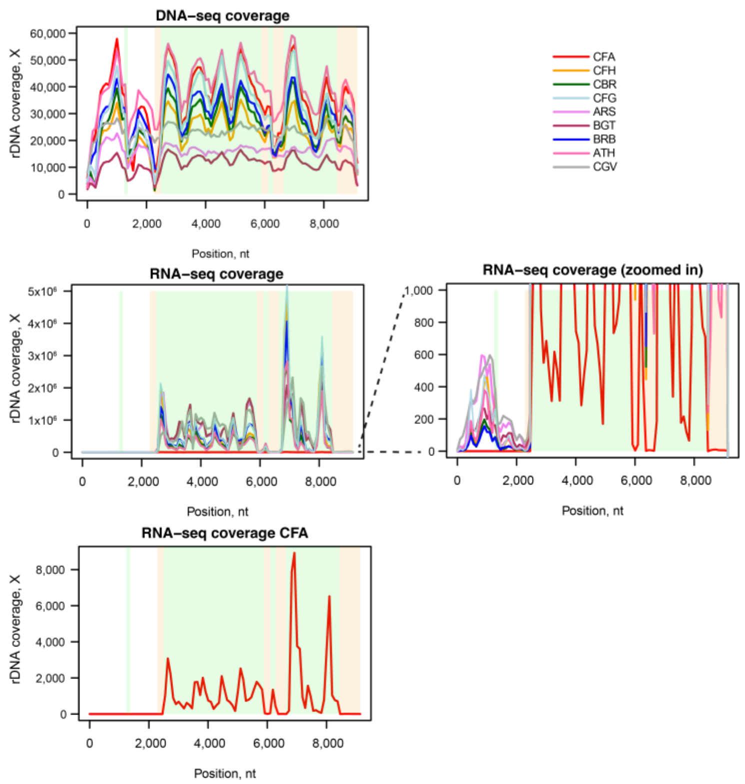

b

Library		Total Read Count
undetermined_library		23 208 839
12267_DNA		11 927 317
12268_DNA		12 051 221
12269_DNA		14 410 096
12270_DNA		12 055 410
12271_DNA		14 671 076
12272_DNA		12 585 743
12273_DNA		11 859 724
12274_DNA		12 797 641
12275_DNA		11 675 009
12267_totalRNA	CFA RNA-seq	167 807
12268_totalRNA		23 112 037
12269_totalRNA		29 918 763
12270_totalRNA		30 632 545
12271_totalRNA		17 787 776
12272_totalRNA		21 149 883
12273_totalRNA		25 329 291
12274_totalRNA		36 546 306
12275_totalRNA		18 912 060

Supplementary Figure 25. Sequencing coverage of the nine isolates with variants in the GAC. a, DNA- and total RNA-seq coverage of the isolates plotted against the rDNA prototype. Each line is a separate isolate. Note the extremely high coverage of the RNA-sequencing data, which indicates that the transcripts are coming from true ribosomal RNA and are expressed in the cell. The lower coverage of the isolate CFA is due to very a low concentration of the sample library in the pool (**b**). Additionally, note that some reads in the RNA-seq data are from *NTS* (non-transcribed sequences), and their very minor fraction suggest they are coming either from the genomic DNA or leaky transcription. Source data are provided as a Source Data file.

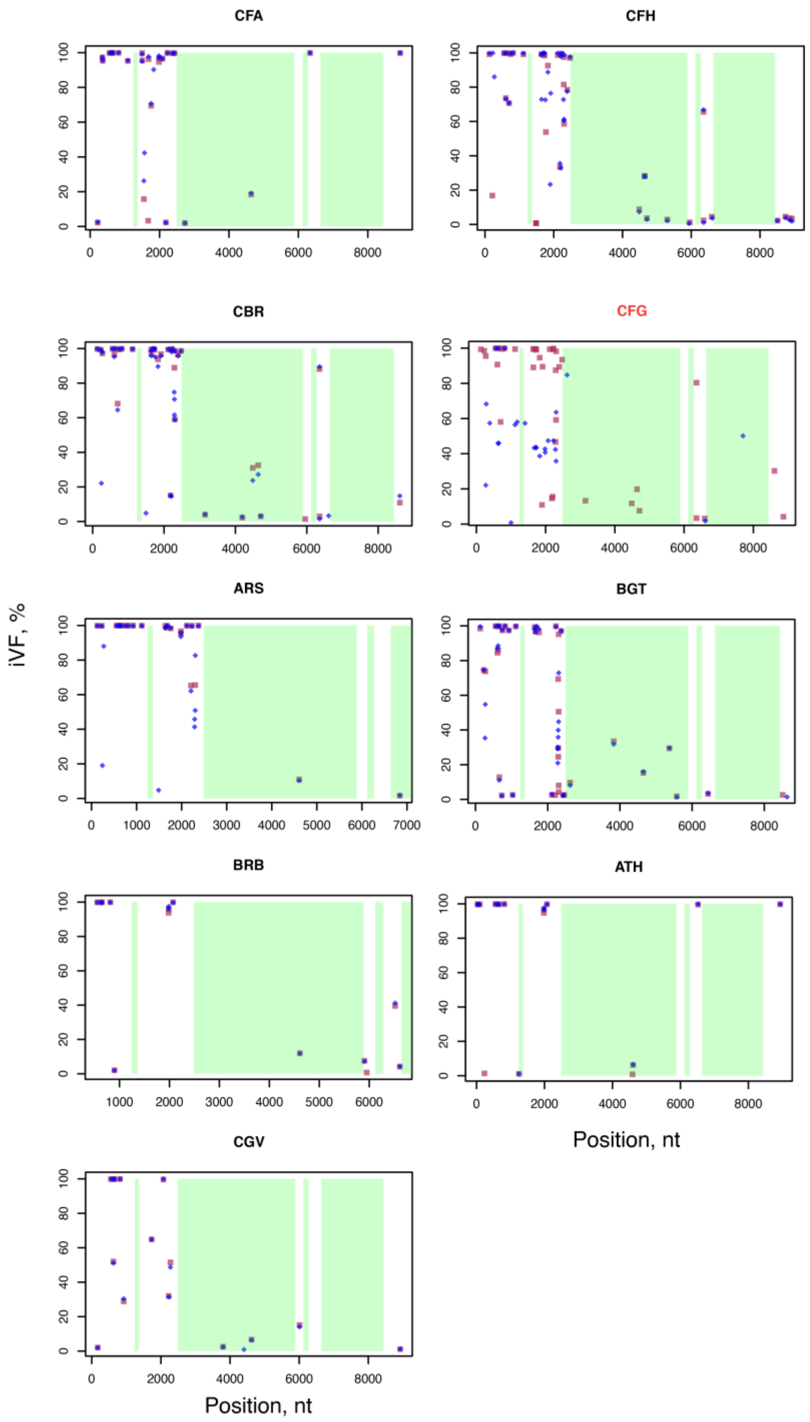

Supplementary Figure 26. Variants detected from the DNA-sequencing data. Red dots indicate variants detected in DNA sequencing data from [2], and blue dots indicate variants detected in DNA sequencing data from the current study (without filtering for high strand bias). The discordant "CFG" isolate is highlighted in red. Source data are provided as a Source Data file.

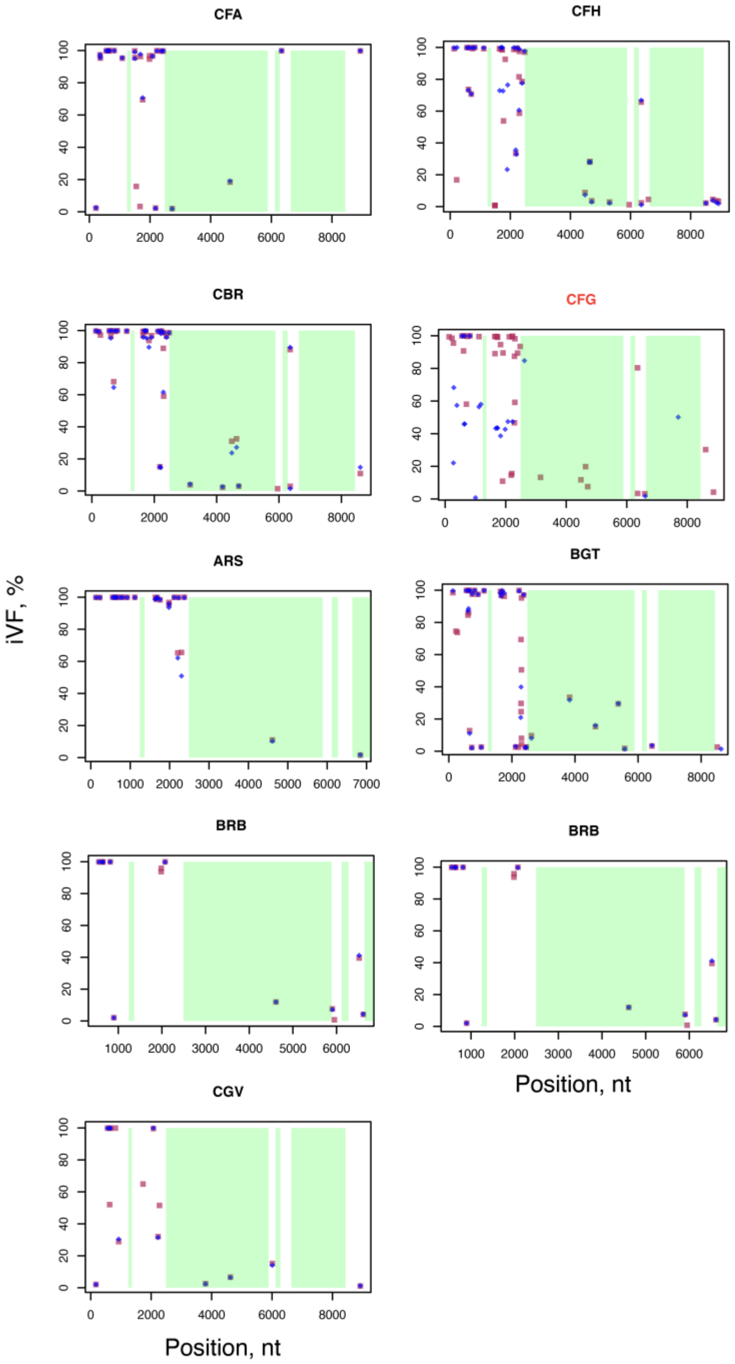

Supplementary Figure 27. Same as Supplementary Figure 26 but with additional filtering for high strand bias. Top 10% of variants with the highest strand bias were removed in each isolate. Some variants in *NTS, ETS* and *ITS* were affected by the implicated strand bias filter, suggesting a trade-off between the decreased false positive rate and increase true positive calls. The discordant “CFG” isolate is highlighted in red. Source data are provided as a Source Data file.

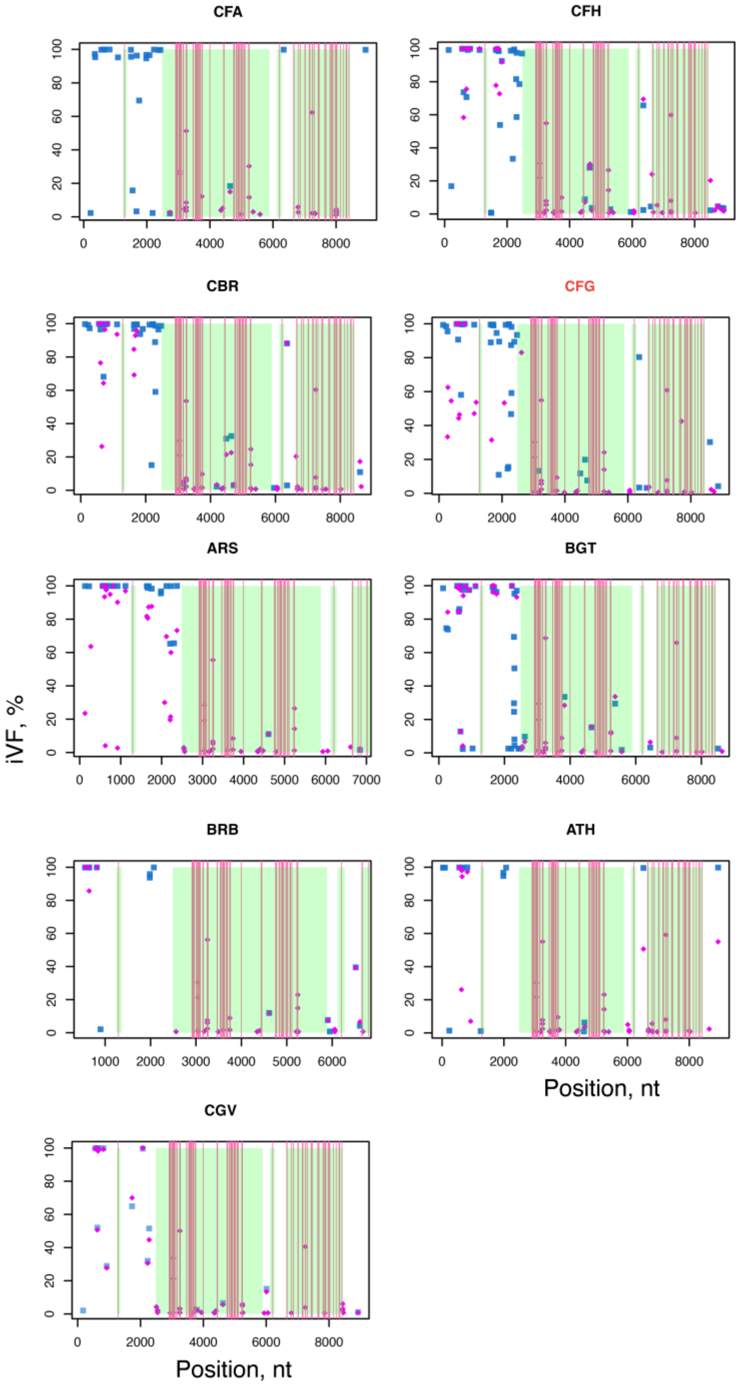

Supplementary Figure 28. Variants detected in the current study. Blue dots are variants detected in the DNA-sequencing data from this study, and purple dots are variants detected in the RNA-sequencing data. Red lines indicate positions of rRNA post-transcriptional modification from [3], which align with consistent false-positive calls from RNA-sequencing data. The presence of variants in *NTS* the in RNA-sequencing data is due to possible DNA contamination or leaky transcription (see **Supplementary Figure 25**). Source data are provided as a Source Data file.

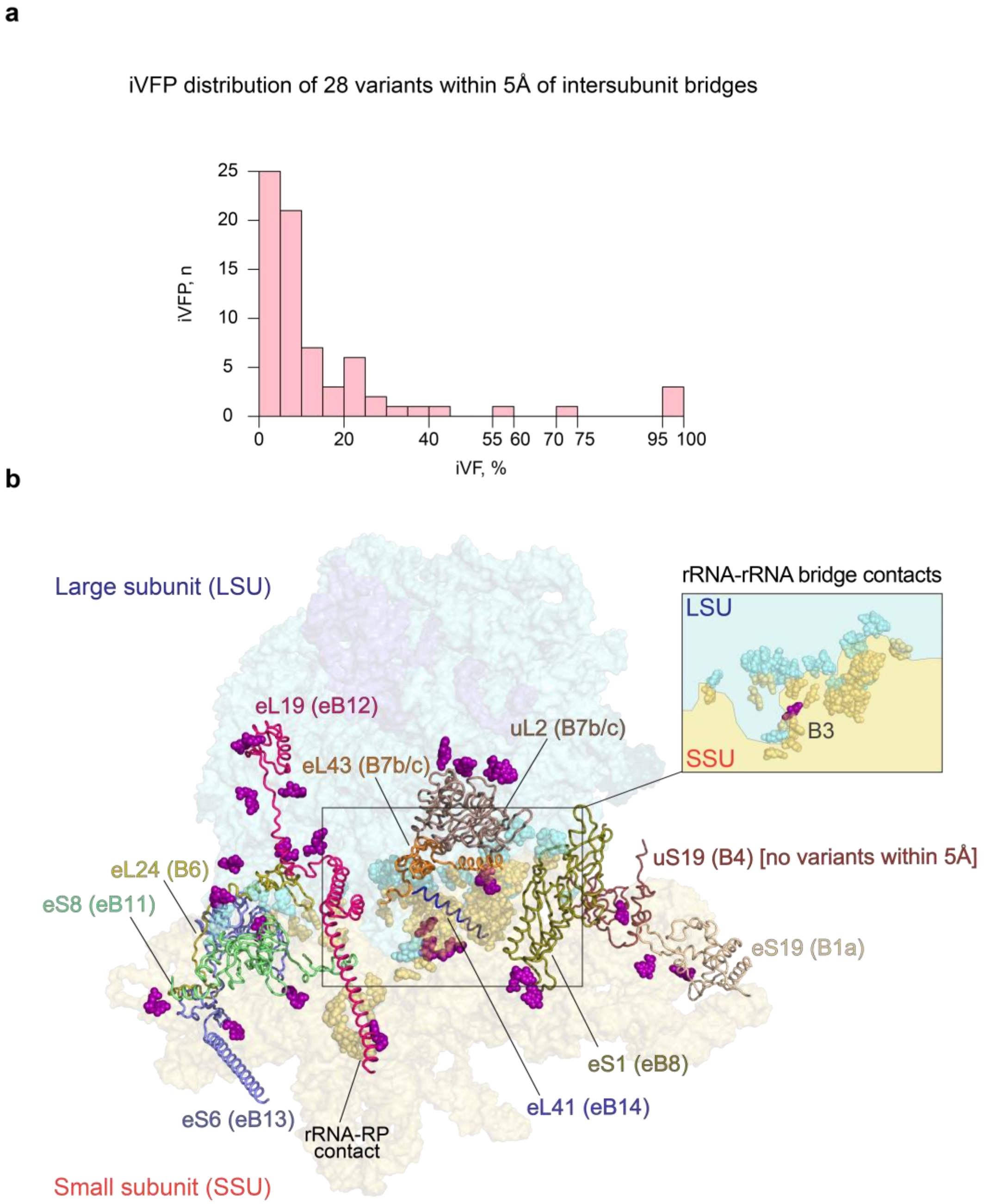

Supplementary Figure 29. Variants are localized in the vicinity of intersubunit bridges. a, iVFP distribution of the 26 variants found within 5Å of the intersubunit bridges. **b**, variants (shown in purple with the spherical representation) within 5Å of the intersubunit bridges components (protein part of a bridge is shown with the backbone representation, rRNA part is show with the transparent spherical representation). Blue outline - large subunit (LSU); yellow outline - small subunit (SSU). Ribosomal protein (RP) names along with the corresponding bridges (in brackets) are indicated. *inset* - bridge contacts mediated by rRNA only. A variant position near bridge B3 is indicated in the inset. Source data are provided as a Source Data file.

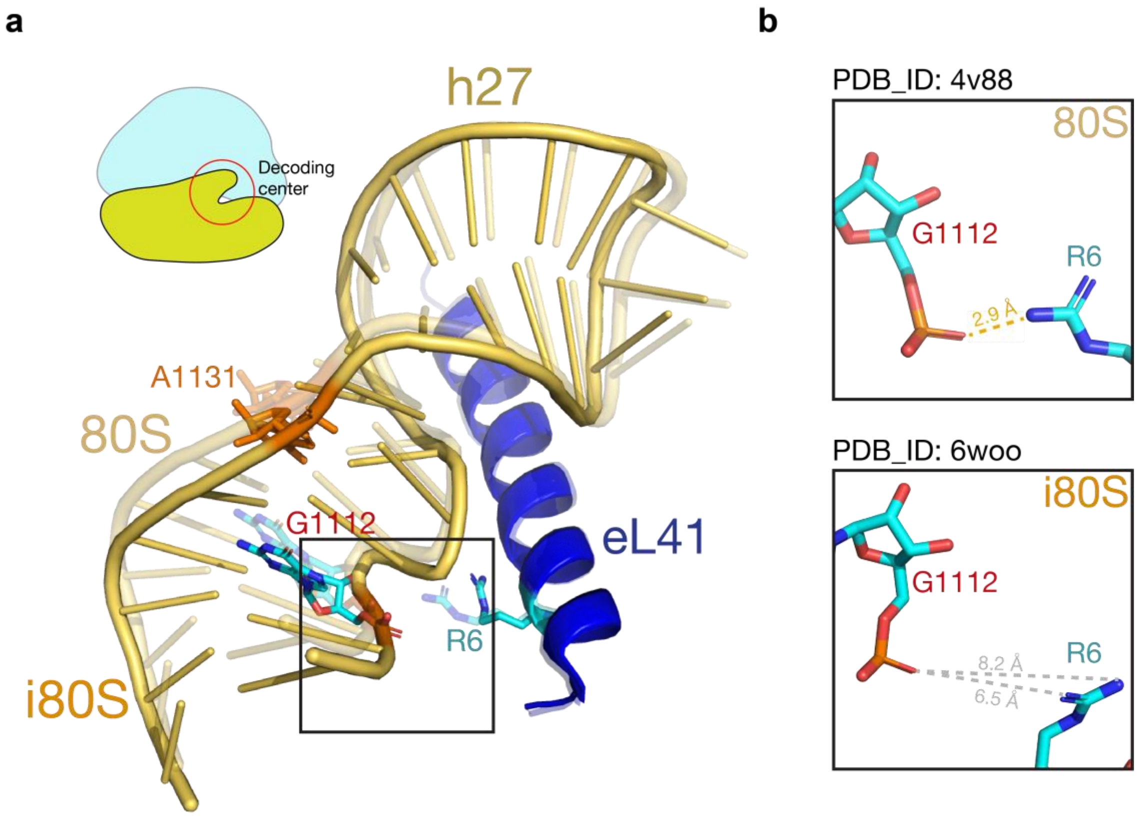

Supplementary Figure 30. Structural alignment of a region near the decoding center. a, 80S ribosome structures at two different stages of translation - non-elongating ("80S", transparent; PDB ID: 4v88 at 3.0Å) and initiating-to-elongating state ("i80S", bold; PDB ID: 6woo at 2.9 Å). The region encompasses helix h27 and polypeptide eL41; the latter is part of intersubunit bridge eB14. Positions at which variants were founds are highlighted (G1112 and A1131; 18S nomenclature). **b**, zoom into the region between G1112 and eL41. The phosphate backbone of G1112 forms a hydrogen bond with R6 of eL41 in the 80S (*top*), while no interaction occurs in the i80S (*bottom*) due to conformational change.

SUPPLEMENTARY REFERENCES

1. de Chiara, M. *et al.* Domestication reprogrammed the budding yeast life cycle. *Nat Ecol Evol* **6**, 448–460 (2022).
2. Peter, J. *et al.* Genome evolution across 1,011 Saccharomyces cerevisiae isolates. *Nature* **556**, 339–344 (2018).
3. Sloan, K. E. *et al.* Tuning the ribosome: The influence of rRNA modification on eukaryotic ribosome biogenesis and function. *RNA Biol* **14**, 1138–1152 (2017).
