# Supplementary material for: Varying strength of selection contributes to the intragenomic diversity of rRNA genes
Source: Nat Commun. 2022 Nov 25;13:7245. doi: 10.1038/s41467-022-34989-w (PMC9700816; doi:10.1038/s41467-022-34989-w)
Supplement: Supplementary file 3 — Description of Additional Supplementary Files [file 41467_2022_34989_MOESM3_ESM.pdf]

Description of Additional Supplementary Files

File Name: Supplementary Data 1
Description: rDNA variants with their frequencies across isolates after filtering #annotation descriptions for POSITION, VF(AF in LoFreq), INDEL, HRUN, QUAL_SCORE, SB, DP and DP4 are as in LoFreq #NAME - Isolate name (from Peter et al., 2018 10.1038/s41586-018- 0030-5) #ACCESSION - SRA accession id (from Peter et al., 2018 10.1038/s41586-018-0030-5) #POSITION - Nucleotide position in the rDNA prototype (S288c reference) #REFERENCE - Nucleotide in the S288C reference at corresponding POSITION #ISOLATE - Variant sequence in analyzed isolate #VF - intragenomic Variant frequency (iVF) #INDEL - indicates if the variant is an indel #HRUN - Homopolymer length to the right of reported indel position #QUAL_SCORE - Quality score #SB - Strand bias #DP - Raw depth #DP4 - Counts for ref-forward bases, ref-reverse, alt-forward and altreverse bases #one_copy_VF - calculated iVF corresponded to a variant in one rDNA copy based on total rDNA copy number in this isolate

File Name: Supplementary Data 2
Description: Calculated Shanonn's entropy at each position in rDNA across all rDNA copies from all isolates pooled together #POSITION - position in the rDNA prototype #A, T, G, C, INDEL - number of sequences with A, T, G, C, and indels at a given position (indels of all sizes are lumped together in INDEL) #H - Shannon's entropy

File Name: Supplementary Data 3
Description: Variant pairs with coherent frequencies across all isolates #NAME - Isolate name (from Peter et al., 2018 10.1038/s41586-018- 0030-5) #ACCESSION - SRA accession id (from Peter et al., 2018 10.1038/s41586-018-0030-5) #POSITION_1 - Nucleotide position in the rDNA prototype for variant 1 in a pair #VARIANT_1 - Sequence of variant 1 in a pair #VF_1 - Intragenomic variant frequency (iVF) of variant 1 #POSITION_2 - Nucleotide position in the rDNA prototype for variant 2 in the pair #VARIANT_2 - Sequence of variant 2 in the pair #VF_2 - Intragenomic variant frequency (iVF) of variant 2

File Name: Supplementary Data 4
Description: Variant pairs with coherent frequencies analyzed by ecological niche #NAME - Isolate name (from Peter et al., 2018 10.1038/s41586-018- 0030-5) #ACCESSION - SRA accession id (from Peter et al., 2018 10.1038/s41586-018-0030-5) #NICHE - ecological origin (from Peter et al., 2018 10.1038/s41586-018-0030-5) #POSITION_1 - Nucleotide position in the rDNA prototype for variant 1 in a pair #VARIANT_1 - Sequence of variant 1 in a pair #VF_1 - Intragenomic variant frequency (iVF) of variant 1 #POSITION_2 - Nucleotide position in the rDNA prototype for variant 2 in the pair #VARIANT_2 - Sequence of variant 2 in the pair #VF_2 - Intragenomic variant frequency (iVF) of variant 2

File Name: Supplementary Data 5
Description: rRNA variant annotation #SUBUNIT - Ribosomal subunit (SSU - small, LSU - large) #RRNA - rRNA #DNA_POSITION - Nucleotide position in the rDNA prototype (S288c reference); rDNA coordinates #DNA_REFERENCE - Nucleotide in the S288C reference at corresponding POSITION; rDNA coordinates #DNA_ISOLATE - Variant sequence in analyzed isolate; rDNA coordinates #RNA_POSITION - Nucleotide position in the prototype (S288c reference); rRNA coordinates #RNA_REFERENCE - Nucleotide in the S288C reference at corresponding POSITION; rRNA coordinates #RNA_ISOLATE - Variant sequence in analyzed isolate; rRNA coordinates #VF - Intragenomic variant frequency (iVF) #SHELL - Subunit shells (from Bernier et al. 2014 10.1039/c3fd00126a) #ES - Expansion segments (from Ben-Shem et al. 2011 10.1126/science.1212642) #RP - Ribosomal proteins (from Ben-Shem et al. 2011 10.1126/science.1212642) #CNE - Presence in eukaryotic conserved nucleotide elements (from Doris et

al. 2015 10.1261/rna.051144.115); defined only for 25S #BRIDGE_WITHIN_5A - Presence within 5 Angstrom of intersubunit bridges (Bridges are from Ben-Shem et al. 2011 10.1126/science.1212642) #INDEL - Indel variants #NAME - Isolate name (from Peter et al., 2018 10.1038/s41586-018- 0030-5) #ACCESSION - SRA accession id (from Peter et al., 2018 10.1038/s41586-018-0030-5)

File Name: Supplementary Data 6
Description:
1) raw_rDNA_var_calls - raw rDNA .vcf files prior to additional filtering
2) Sequencing_Sultanov_etal – raw rDNA .vcf files (for DNA- and total RNA sequencing) generated in this study and the rDNA coverage for each sample
3) rDNA_S288c.fsa - the S288c rDNA copy prototype sequence used in this study
4) rDNA_S288c.bed - annotations associated with the rDNA prototype
5) rDNA_S288c_benchmark.bed - annotations for benchmarking
6) positions_in_homopolymers.txt – nucleotide positions in the S288c rDNA prototype that are embedded in the 10-nt poly(A/T/G/C) sequences.
