# Supplementary material for: Varying strength of selection contributes to the intragenomic diversity of rRNA genes
Source: Nat Commun. 2022 Nov 25;13:7245. doi: 10.1038/s41467-022-34989-w (PMC9700816; doi:10.1038/s41467-022-34989-w)
Supplement: Supplementary file 5 — Reporting Summary [file 41467_2022_34989_MOESM5_ESM.pdf]

nature portfolio

Corresponding author(s): Andreas Hochwagen (ah128@nyu.edu)

Last updated by author(s): Nov 6, 2022

Reporting Summary

Nature Portfolio wishes to improve the reproducibility of the work that we publish. This form provides structure for consistency and transparency in reporting. For further information on Nature Portfolio policies, see our Editorial Policies and the Editorial Policy Checklist.

Statistics

For all statistical analyses, confirm that the following items are present in the figure legend, table legend, main text, or Methods section.

n/a	Confirmed	
☐	☒	The exact sample size (*n*) for each experimental group/condition, given as a discrete number and unit of measurement
☒	☐	A statement on whether measurements were taken from distinct samples or whether the same sample was measured repeatedly
☐	☒	The statistical test(s) used AND whether they are one- or two-sided<br>*Only common tests should be described solely by name; describe more complex techniques in the Methods section.*
☒	☐	A description of all covariates tested
☐	☒	A description of any assumptions or corrections, such as tests of normality and adjustment for multiple comparisons
☐	☒	A full description of the statistical parameters including central tendency (e.g. means) or other basic estimates (e.g. regression coefficient) AND variation (e.g. standard deviation) or associated estimates of uncertainty (e.g. confidence intervals)
☐	☒	For null hypothesis testing, the test statistic (e.g. *F*, *t*, *r*) with confidence intervals, effect sizes, degrees of freedom and *P* value noted<br>*Give P values as exact values whenever suitable.*
☒	☐	For Bayesian analysis, information on the choice of priors and Markov chain Monte Carlo settings
☒	☐	For hierarchical and complex designs, identification of the appropriate level for tests and full reporting of outcomes
☐	☒	Estimates of effect sizes (e.g. Cohen's *d*, Pearson's *r*), indicating how they were calculated

Our web collection on statistics for biologists contains articles on many of the points above.

Software and code

Policy information about availability of computer code

Data collection	Publicly available datasets were downloaded using sra-tools v.2.10.5
Data analysis	Alignment and variant call: bowtie2 v.2.3.4.3, samtools v.1.3.1, LoFreq v.2.1.3.1, bedtools v.2.27.1<br>Pipeline performance: NEAT-genReads v.2.0 (using python v.2.7)<br>Analysis: R v.4.0.3<br>Structural analysis: PyMOL v.2.4.1 (using python v.3.7.9)<br>Other: IGV v.2.9.1<br>Computer scripts generated in this study are available at https://github.com/hochwagenlab/rDNA

For manuscripts utilizing custom algorithms or software that are central to the research but not yet described in published literature, software must be made available to editors and reviewers. We strongly encourage code deposition in a community repository (e.g. GitHub). See the Nature Portfolio guidelines for submitting code & software for further information.

Data

Policy information about availability of data

All manuscripts must include a data availability statement. This statement should provide the following information, where applicable:

- Accession codes, unique identifiers, or web links for publicly available datasets
- A description of any restrictions on data availability
- For clinical datasets or third party data, please ensure that the statement adheres to our policy

Sequence data generated in this study have been deposited in the SRA database under accession code "PRJNA867718 [https://www.ncbi.nlm.nih.gov/bioproject/PRJNA867718/]". The data generated in this study are provided in the Supplementary Information/Source Data file. Supplementary Data 1 is a tab-delimited table of filtered rDNA variants with their intragenomic frequencies. Supplementary Data 2 is a tab-delimited table of calculated Shannon's entropy values at each position of the rDNA. Supplementary Data 3 is a tab-delimited table of variant pairs with consistently similar frequencies across all isolates. Supplementary Data 4 is a tab-delimited table of variant pairs with consistently similar frequencies calculated by niche. Supplementary Data 5 is a tab-delimited table with annotations of variants in rRNA genes. Source data and additional files provided with this paper: 1) raw_rDNA_var_calls - raw rDNA .vcf files prior to additional filtering; 2) Sequencing_Sultanov_etal – raw rDNA .vcf files (for DNA- and total RNA sequencing) generated in this study and the rDNA coverage for each sample; 3) rDNA_S288c.fsa - the S288c rDNA copy prototype sequence used in this study; 4) rDNA_S288c.bed - annotations associated with the rDNA prototype; 5) rDNA_S288c_benchmark.bed - annotations for benchmarking; 6) positions_in_homopolymers.txt – nucleotide positions in the S288c rDNA prototype that are embedded in the 10-nt poly(A/T/G/C) sequences. Other previously published sequencing data used in this study are available in the SRA database under accession codes "ERP014555 [https://www.ncbi.nlm.nih.gov/sra/?term=ERP014555]", "SRR4074258 [https://www.ncbi.nlm.nih.gov/sra/?term=SRR4074258]", and "SRR4074255 [https://www.ncbi.nlm.nih.gov/sra/?term=SRR4074255]"). Previously published structures used in this study are available in the PDB database under accession codes "4v88 [https://www.wwpdb.org/pdb?id=pdb_00004v88]" and "6woo [https://www.wwpdb.org/pdb?id=pdb_00006woo]"

Human research participants

Policy information about studies involving human research participants and Sex and Gender in Research.

Reporting on sex and gender	NA
Population characteristics	NA
Recruitment	NA
Ethics oversight	NA

Note that full information on the approval of the study protocol must also be provided in the manuscript.

Field-specific reporting

Please select the one below that is the best fit for your research. If you are not sure, read the appropriate sections before making your selection.

☒ Life sciences ☐ Behavioural & social sciences ☐ Ecological, evolutionary & environmental sciences

For a reference copy of the document with all sections, see nature.com/documents/nr-reporting-summary-flat.pdf

Life sciences study design

All studies must disclose on these points even when the disclosure is negative.

Sample size	No statistical methods were used to determine sample size.
Data exclusions	Read alignment and variant call: to reduce the rate of false positive calls, entries were excluded EXCEPT if: 1) nucleotide position > 10 and < 9100, 2) iVF > 0.005, 3) homopolymer run < 4, 4) GC content < 0.6 in long indels (> 5 nt) in either reference or alternative sequences, 5) variants are outside of 10-nt stretches of poly A/T/G/C sequences, 6) the minimal iVF > theoretical one copy iVFs computed for each isolate separately, and 7) variants with strand bias below 144.<br>Deduplicated isolates and the respective used accessions in this study (name - accession) : AFV - ERR1308598, AGB - ERR1308892, AGC - ERR1309000; AGF - ERR1309486; AGG - ERR1308790; AGH - ERR1309035; AGI - ERR1308800; AGK - ERR1308845; AGM - ERR1309110; AGR - ERR1309258; ARS - ERR1308992; BAM - ERR1308775; BFN - ERR1308958; BGN - ERR1308908; BGP - ERR1308957; CKR - ERR1309527; CLC - ERR1308723; CLD - ERR1308823; CNE - ERR1308656<br>rRNA analysis: 247 nucleotides in LSU rRNA and 19 nucleotides in SSU rRNA were excluded from analysis (due to their absence in the used crystal structure of the ribosome) where applicable (distribution of iVFPs across shells; across ESs and RP contacts)
Replication	Replication was not performed in this study
Randomization	Randomization is not applicable; no treatment/control experiments were conducted

Blinding | Blinding is not applicable

Reporting for specific materials, systems and methods

We require information from authors about some types of materials, experimental systems and methods used in many studies. Here, indicate whether each material, system or method listed is relevant to your study. If you are not sure if a list item applies to your research, read the appropriate section before selecting a response.

Materials & experimental systems

n/a	Involved in the study	
☒	☐	Antibodies
☒	☐	Eukaryotic cell lines
☒	☐	Palaeontology and archaeology
☒	☐	Animals and other organisms
☒	☐	Clinical data
☒	☐	Dual use research of concern

Methods

n/a	Involved in the study	
☒	☐	ChIP-seq
☒	☐	Flow cytometry
☒	☐	MRI-based neuroimaging
